# Polyketide Derivatives in the Resistance of *Gerbera hybrida* to Powdery Mildew

**DOI:** 10.3389/fpls.2021.790907

**Published:** 2022-01-06

**Authors:** Anna Mascellani, Kirsten Leiss, Johanna Bac-Molenaar, Milan Malanik, Petr Marsik, Estuardo Hernandez Olesinski, Jan Tauchen, Pavel Kloucek, Karel Smejkal, Jaroslav Havlik

**Affiliations:** ^1^Department of Food Science, Faculty of Agrobiology, Food and Natural Resources, Czech University of Life Sciences Prague, Prague, Czechia; ^2^Business Unit Greenhouse Horticulture, Wageningen University & Research, Bleiswijk, Netherlands; ^3^Department of Natural Drugs, Faculty of Pharmacy, Masaryk University, Brno, Czechia

**Keywords:** *Gerbera hybrida*, powdery mildew resistance, ^1^H NMR, decision tree, polyketides

## Abstract

Powdery mildew is a common disease affecting the commercial production of gerbera flowers (*Gerbera hybrida*, Asteraceae). Some varieties show a certain degree of resistance to it. Our objective was to identify biomarkers of resistance to powdery mildew using an ^1^H nuclear magnetic resonance spectroscopy and chemometrics approach in a complex, fully factorial experiment to suggest a target for selection and breeding. Resistant varieties were found to differ from those that were susceptible in the metabolites of the polyketide pathway, such as gerberin, parasorboside, and gerberinside. A new compound probably involved in resistance, 5-hydroxyhexanoic acid 3-*O*-*β*-D-glucoside, was described for the first time. A decision tree model was built to distinguish resistant varieties, with an accuracy of 57.7%, sensitivity of 72%, and specificity of 44.44% in an independent test. Our results suggest the mechanism of resistance to powdery mildew in gerbera and provide a potential tool for resistance screening in breeding programs.

## Introduction

Plants as sessile organisms cannot escape from unfavorable conditions in their surroundings. For this reason, they have acquired the ability to produce a remarkable diversity of low-molecular-weight compounds to protect themselves. They produce a vast range of so-called secondary metabolites, specifically adapted to combat exposure to pathogens or herbivores both above and below the ground (Pichersky and Gang, 2000; Sirikantaramas et al., 2008). Those defensive compounds are subjected to changes as the plant continues to evolve and adapt. Differences in the content of such chemicals between plants are a key factor in the study of plant resistance, an important part of the integrated management of disease (Leiss et al., 2009). More than one compound is usually involved in such biological processes, providing additional if not synergistic effects and reducing the chance that pathogens and herbivores might develop resistance.

Metabolomics is a suitable tool for the chemical characterization of compounds involved in plant resistance, allowing simultaneous detection of a wide range of compounds (Kim et al., 2010). NMR spectroscopy can identify and quantify compounds quickly and reproducibly with little sample preparation (Allwood et al., 2008; Verpoorte et al., 2008; Kim et al., 2010). Chemometric tools for data mining encourage the exploratory use of NMR for metabolic fingerprinting (Winning et al., 2008). NMR has been successfully applied to study metabolites associated with pathogen (Kashif et al., 2009) and herbivore (Leiss et al., 2009, 2011, 2013) resistance. In comparison with the most common analytical metabolomics techniques, NMR is characterized by its high reproducibility. Throughout the years, chromatographic methods and mass spectrometry have shown to be affected by intra- and inter-day variability and inconsistency in reproducing the same results in another lab. Unlike chromatographic methods, NMR does not require continual calibration curves for each compound to be quantified, but instead can rely on in-house databases (Verpoorte et al., 2007). NMR is very well-suited for high-throughput methodology, including the possibility of robotization. A 3-min analysis can provide quantitative data. Despite the limited number of compounds detected in an extract and that certain minor compounds might not be observed, the major trends are clear (Verpoorte et al., 2007); NMR can be well-integrated with other spectroscopic or spectrometric techniques to identify metabolites and elucidate their structures (Wolfender et al., 2003; Kim et al., 2010).

Gerbera is one of the ten most economically important flower crops in Europe (Teeri et al., 2006) and the United States (USDA, 2019). It is a popular ornamental plant used as a cut flower, potted plant, or landscape bedding plant to decorate gardens, terraces, and patios. Cultivated gerberas are derived from crosses between the wild species *Gerbera jamesonii* Bolus ex. Hook f. and *Gerbera viridifolia* (DC.) Sch. Bip. and have been given the provisional name *Gerbera hybrida*. An important objective for gerbera breeders is to improve the resistance of the varieties to major fungal diseases, particularly powdery mildew (Kloos et al., 2005b; Deng and Harbaugh, 2010). Powdery mildew, a key economically important fungal disease of gerbera, is caused by *Podosphaera xanthii* (Castagne) U. Braun and N. Shishkoff (syn. *Sphaerotheca fuliginea*, formerly *Sphaerotheca fusca* Blumer). This disease is considered to be the most common and destructive disease of gerbera in commercial production and landscape use, affecting all parts of the plant, leading to substantially reduced growth and loss of quality (Kloos et al., 2005b; Song and Deng, 2013; Ghani and Sharma, 2019). Symptoms of powdery mildew consist of white spots on the upper surfaces of the leaf, which gradually enlarge to form a white powder-like mat that can spread to the flowers and the stems. The genetic basis of resistance to powdery mildew in gerbera is poorly understood; putative sequences and markers have been studied (Song et al., 2012; Song and Deng, 2013). The dominant allele determining powdery mildew resistance, *Pmr1*, has been identified in a wild type of gerbera (Kloos et al., 2005b). Recently, resistance and susceptibility genes in gerbera associated with powdery mildew were revealed (Bhattarai et al., 2020). However, this study is only based on two breeding lines, a resistant and a susceptible one, respectively. Gerbera is considered a model plant for plant reproduction exhibiting secondary metabolism of anthocyanins and polyketides (Eckermann et al., 1998; Koskela et al., 2001; Kloos et al., 2005a; Teeri et al., 2006; Broholm et al., 2010; Ruokolainen et al., 2010; Bashandy et al., 2015; Pietiäinen et al., 2016).

Information about resistance to powdery mildew in gerbera is scarce and little is known about the underlying mechanisms. Thus, in this study, we looked at differences in the composition of metabolites, both identities and concentrations, between resistant and susceptible varieties and aimed at providing a tool for the prediction of the resistance to powdery mildew based on the metabolome. An untargeted NMR-based metabolomics approach was applied to a set of 120 samples, including eight different varieties, four classified as resistant and four as susceptible. Standard and mini gerbera and five replicates of young and old leaves were included. Compounds for which the peaks in the NMR spectrum differed between the groups were further isolated and their structures elucidated by means of semi-preparative HPLC, LC-MS, and NMR, and quantified by ^1^H NMR to build a decision tree enabling rapid screening for the selection of varieties and systematic plant breeding.

## Materials and Methods

### Plant Material

**Figure 1:**
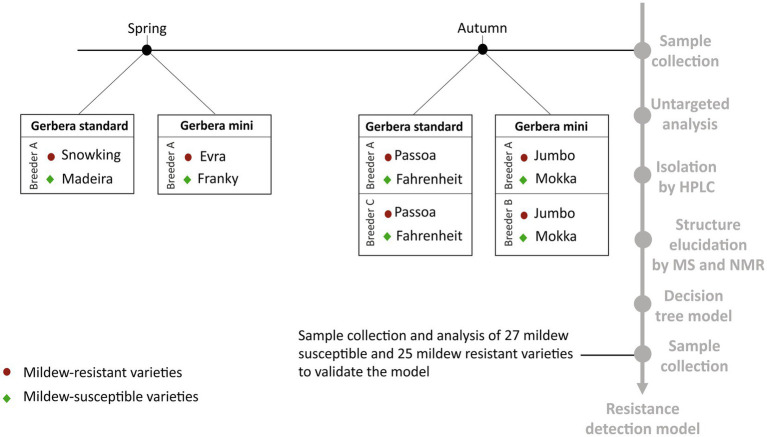
Timeline diagram of the study design.

Eight varieties of *G. hybrida* grown by three Dutch breeders were used for this study (Figure 1). The plants were described as either resistant or susceptible to powdery mildew based on the empirical experience of the breeders. Half of the samples were gerbera mini varieties, of which two (varieties Evra and Jumbo) were resistant to powdery mildew and two were susceptible (Franky and Mokka). The other four were standard varieties of gerbera, of which two (varieties Snowking and Passoa) were resistant to powdery mildew and two were susceptible (Madeira and Fahrenheit). The samples were collected in spring and autumn (Figure 1). For each variety, young and old leaves were collected separately in five biological replicates, each consisting of 3 old or 5 young, pooled leaves. From each individual plant, the third (young) leaf from above and each third (old) leaf from below was sampled for the analysis. This resulted in a total of 120 samples. A second sample set was collected for the external validation of the model (Figure 1). A pooled sample of 5 leaves from different plants was collected from 27 mildew-susceptible and 25 mildew-resistant varieties (Supplementary Table S2), based on the empirical experience of the breeders. It included the varieties used to build the models and other varieties not previously included, the same varieties from different breeders were included. The plant material was immediately frozen in liquid nitrogen upon harvesting and freeze-dried. After freeze-drying, samples were ground using a laboratory grinder IKA A 11 basic Analytical Grinder Mill and stored at −80°C until extraction.

### Chemicals

All chemicals and reagents used were of analytical grade. Potassium dihydrogen phosphate (99%, KH_2_PO_4_), deuterium oxide (99.9%, D_2_O), methanol-*d*_4_ (>99.8%, MeOD), and methanol were purchased from VWR (Radnor, PA, United States). Sodium deuteroxide 40% w/v solution in D_2_O (99.5%, NaOD) was obtained from Alfa Aesar (Kandel, Germany). 3-(Trimethylsilyl) propionic-2,2,3,3-*d*_4_ acid sodium salt (99%, TMSP) was obtained from Sigma-Aldrich (St. Louis, MO, United States). A Millipore Direct-Q^®^ 3 UV Water Purification System (Millipore Corp., Bedford, MA, United States) or (Merck KGaA, Darmstadt, Germany) for ultrapure water was used throughout the study.

### Extraction of Plant Material and Acquisition of NMR Data

Each 50 mg of the finely ground leaves, were extracted in MeOD-D_2_O (1:1, v/v) as previously described (Kim et al., 2010; Mascellani et al., 2021). All spectra were recorded on a Bruker Avance III spectrometer equipped with a broad band fluorine observation (BBFO) SmartProbe^™^ with z-axis gradients (Bruker BioSpin GmbH, Rheinstetten, Germany), operating at the ^1^H NMR frequency of 500.23 MHz. All ^1^H NMR spectra were acquired and processed under the same conditions (Mascellani et al., 2021). All samples were calibrated to the internal standard TMSP at 0.0 ppm, subject to exponential apodization of 0.3 Hz, and phase- and baseline corrections were made using Mnova software, version 14.1.0 (Mestrelab Research, S.L., Santiago de Compostela, Spain).

### Data Analysis

Spectral intensities of the ^1^H-NMR spectra were scaled to the intensity of the internal standard [TMSP, 0.01% (w/v)] and reduced to spectral bins of 0.04-ppm by the sum of data points corresponding to the region of δ_H_ 0.02 to δ_H_ 10.0 ppm. The regions corresponding to water (δ_H_ 4.70–5.00 ppm) and methanol (δ_H_ 3.28–3.40 ppm) were excluded from the analysis. A script built in-house was used for data reduction. PCA and OPLS-DA were performed using the package MetaboAnalystR (Pang et al., 2020) version 3.0.2 in R 4.0.0 (R Core Team, 2020). PQN, log transformation, and auto-scaling were applied for PCA and OPLS-DA. The OPLS-DA model was validated by R^2^Y and Q^2^ metrics using the permutation method through 100 applications, to describe the percentage of variation explained by the model and its predictive ability, respectively. Graphs were constructed using the package ggplot2 (Wickham, 2016) v. 3.3.3 in R 4.0.0 (R Core Team, 2020). A two-tailed unpaired *t*-test comparison (no correction for multiple comparison) was performed for each 0.04 ppm bin. Color-coded graphs were generated from an in-house script in MATLAB^®^ R2020a (The Mathworks). The signals from the most significant [*p*(corr) > |0.5| and/or *p* < 0.05] bins results were identified and quantified.

### Extraction and Isolation to Identify the Compounds

The discriminating NMR signals between the two varieties were isolated by NMR-guided semi-preparative HPLC. To identify the metabolites, pooled samples of resistant leaves were extracted by decoction for 15 min and filtered. The resulting extract with an approx. concentration of 5 mg/ml of dry weight was used for fractionation collection using an HPLC (UltiMate^™^ 3000 system, Thermo Fisher Scientific, Waltham, MA, United States) equipped with a Dionex Ultimate 3000 UV detector and fraction collector (Thermo Fisher Scientific, Waltham, MA, United States). The chromatographic separation was carried out on a Triart C18 column (5 μm, 250 × 4.6 mm; YMC CO., LTD., Kyoto, Japan) coupled with Polar C18 SecurityGuard Cartridges (Phenomenex, Torrance, CA, United States) at 35°C, with a flow rate of 0.8 ml/min and an injection volume of 100 μl. The mobile phase consisted of (A) Milli-Q water and (B) methanol. The gradient was as follows: 0% B isocratically for 5 min followed by increase to 100% B over 10 min, a hold for 5 min, and a subsequent return to the initial conditions. Fractions were collected every 24 s, resulting in a total of 48 fractions. Multiple injections were pooled. The fractions collected were freeze-dried, and each residue was resuspended in MeOD and D_2_O (1:1, v/v) for NMR fast screening. Selected fractions were pooled and purified by a second HPLC fractionation with the same conditions as described above.

### Identification of the Isolated Compounds

The acquired 2D NMR spectra consisted of DQF-COSY, HMBC, and HSQC with common standard settings (Supplementary Table S3). NMR spectra were recorded on the spectrometer previously described. The spectra were processed with Mnova software version 14.1.0 (Mestrelab Research). The MS/MS spectra were acquired using a qualitative tandem liquid chromatography-quadrupole time-of-flight mass spectrometry (LC-QTOF-MS/MS) system consisting of Dionex Ultimate 3000 ultra-high performance liquid chromatography chromatograph (Thermo Fisher Scientific, Waltham, MA, United States) coupled with an Impact II quadrupole time-of-flight mass spectrometer with high-resolution accurate mass (Bruker Daltonics, Bremen, Germany), equipped with an electrospray ionization source in both positive and negative ionization modes. Liquid chromatography separations and the ionization parameters for mass spectrometry were performed using the conditions described in Supplementary Table S4. The chromatography was controlled by Chromeleon Xpress link (Thermo Fisher Scientific, Waltham, MA, United States). The MS/MS fragmentation spectra were acquired by O-Tof Control 4.0 and HyStar 3.2 software (Bruker Daltonik, Bremen, Germany). The spectra were processed using Compass DataAnalysis 4.3 (Bruker Daltonik, Bremen, Germany). The fragments were analyzed using Mass Frontier 7.0.5.9 SR3 (High Chem Ltd., Bratislava, Slovakia) and Thermo Excalibur 3.0.63 (Thermo Fisher Scientific, Waltham, MA, United States) software. The exact masses obtained were searched using Bruker Compound Crawler 3.0 databases (Bruker Daltonik, Bremen, Germany) and their fragmentation spectra were compared with MetFrag free online software results.

### Quantification of Metabolites

The peak areas of selected ^1^H NMR signals belonging to the target compounds, and the peak area of TMSP, were integrated manually for all of the samples and the metabolites were quantified by a ratio analysis normalized to the number of protons (Leiss et al., 2009). These quantitative data were used to test differences in the concentration between varieties and between leaf ages by a two-tailed unpaired *t*-test and also to build a decision tree model. The criterion for statistical significance was a probability value of *p* ≤ 0.05.

### Statistical Modeling

The concentrations of the targeted compounds used to build a conditional interference tree were chosen using the package party (Hothorn et al., 2006) v. 1.3-5 in R version 4.0.0. Of the samples, 70% were used for training and the remaining 30% as testing subsets. Accuracy, sensitivity, and specificity were used to evaluate the performance of the decision trees based on the confusion matrix. Accuracy is the probability that a sample is correctly predicted. Sensitivity constitutes the probability that a resistant sample is correctly predicted to be resistant, specificity expresses the probability that a susceptible sample has been correctly identified as susceptible. The model was successively validated with the second dataset collected to evaluate its performance for the applications in breeding.

## Results

### Data Reduction

**Figure 2:**
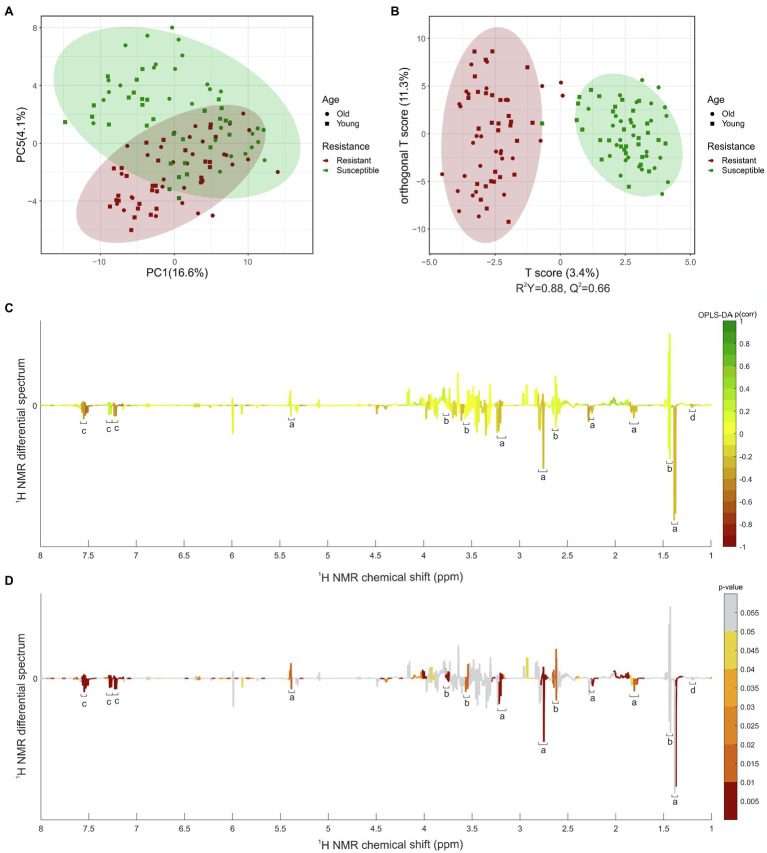
Univariate and multivariate statistical analysis of leaves extracted in MeOD-D_2_O (1:1, v/v). **(A)** PCA scores plot. **(B)** OPLS-DA scores plot. **(C)** OPLS-DA coefficient plot based on the ^1^H NMR differential spectrum (mean susceptible spectrum, *N* = 60, minus mean resistant spectrum, *N* = 60). **(D)** *t*-test coefficient plot based on the ^1^H NMR differential spectrum (mean susceptible spectrum, *n* = 60, minus mean resistant spectrum, *n* = 60). ^a^Parasorboside, ^b^gerberin, ^c^gerberinside, ^d^5-hydroxyhexanoic acid 3-*O*-*β*-D-glucoside are shown. Peaks in the positive direction indicate metabolites more abundant in varieties susceptible to mildew. Metabolites more abundant in leaves of varieties resistant to mildew are shown as peaks in the negative direction. The color bar indicates the correlation of the bins with the predicting variation **(C)** and value of *p* **(D)**. The *filled red circle* represents the 95% confidence region of varieties resistant to mildew and the *filled green circle* the 95% confidence region of varieties susceptible to mildew. Filled squares represent the young leaves and filled dots the old leaves of varieties resistant (red) or susceptible (green) to mildew.

Application of the PCA model resulted in eight principal components explaining 50.3% of the total variance. Intergroup separation between the resistant and susceptible varieties occurred across the principal component 5, representing 4.1% of the variation (Figure 2A). Relatively dispersed mildew-resistant and -susceptible samples were observed, reflecting the varied character of the samples. Difference in the ages of the leaves between samples seemed to be relatively unimportant. To explore the segregation between the two classes concerned, a supervised OPLS-DA model was compiled (Figure 2B) to distinguish resistant varieties from these which susceptible. It showed a clear separation between mildew-resistant and -susceptible varieties, with an R^2^Y value of 0.88 and a Q^2^ value of 0.66, suggesting a strong prediction. An additional set of *t*-tests comparing samples (no correction for multiple comparisons was made) from resistant and susceptible varieties was performed (*p* < 0.05). Graphical projection of *p*(corr) using the OPLS-DA and value of *p* values from the *t*-test (Figures 2C, D) indicated the signals contributing most to the class discrimination. Combining multivariate and univariate statistical analyses showed that the characteristic metabolites of resistant varieties had stronger proton resonance signals for bins centered at δ_H_ 7.50 ppm [*p* ≤ 0.01, *p*(corr) = −0.55], δ_H_ 7.46 ppm [*p* ≤ 0.01, *p*(corr) = −0.50], δ_H_ 7.18 ppm [*p* ≤ 0.01, *p*(corr) = −0.48], δ_H_ 7.14 ppm [*p* ≤ 0.01, *p*(corr) = −0.56], δ_H_ 5.94 ppm [*p* ≤ 0.01, *p*(corr) = −0.55], δ_H_ 5.38 ppm (*p* ≤ 0.01), and δ_H_ 1.34 ppm [*p* ≤ 0.01, *p*(corr) = −0.21]. These signals were selected as candidates for further investigating and quantification. Additionally, we included signals at δ_H_ 1.44 ppm, and 1.20 ppm in the comparisons because they were structurally similar to the candidate signal at δ_H_ 1.34 ppm in the centered bin.

### Identification of Metabolites

The candidate signals chosen as markers of resistance to mildew were isolated and assigned to the four compounds gerberin, parasorboside, gerberinside, and 5-hydroxyhexanoic acid 3-O-*β*-D-glucoside, using MS/MS and NMR (Table 1, Supplementary Figures S1–S16). The resonance signals observed for these compounds corresponded to the signals in the bins described above, as illustrated in Figure 3.

**Table 1:** ^1^H and ^13^C NMR spectroscopic assignments in ppm [at 500 and 125 MHz, MeOD-D_2_O (1:1, v/v)] and LC-QTOF-MS/MS assignments of compounds from fractionation of *Gerbera hybrida* aqueous extract.

	LC-QTOF-MS/MS				^1^H and ^13^C NMR		
Compound name & structure	Retention time (conditions)	m/z [M+H]^+^ (formula, calculated mass)^a^	m/z [M−H]^−^ (formula, calculated mass)	Formula	Pos.	*δ*_H_ (mult; J in Hz) ppm	*δ*_C_
5-hydroxyhexanoic acid 3-*O*-*β*-D-glucoside 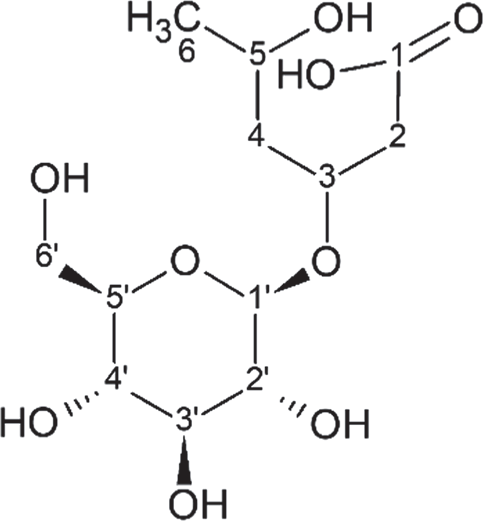	3.6 min (0.2% formic acid and methanol)	311.1336 (C_12_H_23_O_9_, calcd 311.1342)	309.1197 (C_12_H_21_O_9_, calcd 309.1186)	C_12_H_22_O_9_	1	–	176.7^c^
					2	2.46 (qd, 14.4, 14.4, 14.4, 6.3)	43.7
					3	4.23 (m)	77.1
					4	1.65 (dt, 14.2, 5.4) 1.87 (m)	44.1
					5	4.00 (m)	66.8
					6	1.21 (d, 6.2)	22.6
					1′	4.52 (d, 7.9)	70.1
					2′	3.21 (dd, 7.9, 9.2)	74.2
					3′	3.46 (dd, 9.2, 8.6)	76.8
					4′	3.35 (dd, 8.6, 9.8)	70.0
					5′	3.40 (ddd, 9.8, 5.5, 2.1)	76.8
					6′	3.89 (dd, 12.1, 2.1) 3.71 (dd, 12.1, 5.5)	61.6
Gerberin 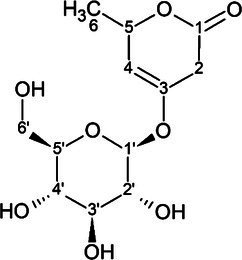	3.9 min (0.2% formic acid and methanol)	291.1071 (C_12_H_19_O_8_, calcd 291.1079)	289.0932 (C_12_H_17_O_8_, calcd 289.0923)	C_12_H_18_O_8_	1	–	180.3^c^
					2	5.40 (d, 1.3)	92.8
					3	–	171.6^c^
					4	2.60 (dd, 1.3 17.5) 2.64 (dd, 4.7, 17.5)	32.1
					5	4.59^c^	72.8
					6	1.45 (d, 6.3)	18.2
					1′	5.11(d, 7.7)	97.5
					2′	3.50 (dd, 7.7, 9.3)	71.5
					3′	3.56 (m)	75.2
					4′	3.45 (m)	68.2
					5′	3.56 (m)	75.2
					6′	3.73 (dd, 5.4, 12.4) 3.90 (dd, 2.2, 12.4)	59.6
Parasorboside 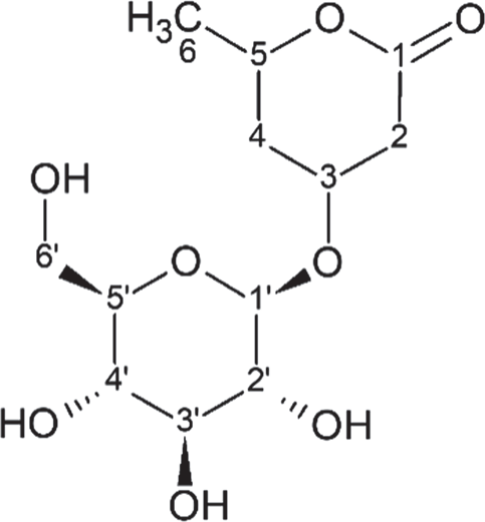	4.0 min (0.2% formic acid and methanol)	293.1225 (C_12_H_21_O_8_, calcd 293.1236)	291.1086 (C_12_H_19_O_8_, calcd 291.1080)	C_12_H_20_O_8_	1	–	^b^
					2	2.80 (m)	33.8
					3	4.41 (m)	69.7
					4	2.26 (m) 1.81 (ddd, 2.9, 11.3, 14.4)	33.8
					5	4.91^c^	73.5
					6	1.39 (d, 6.4)	18.8
					1′	4.50 (d, 7.8)	100.5
					2′	3.22 (dd, 7.8, 9.3)	72.2
					3′	3.45 (m)	75.8
					4′	3.34 (dd, 8.8, 9.8)	68.7
					5′	3.40 (ddd, 2.2, 5.8, 9.8)	75.1
					6′	3.88 (dd, 2.2, 12.3) 3.68 (dd, 5.8, 12.3)	59.6
Gerberinside 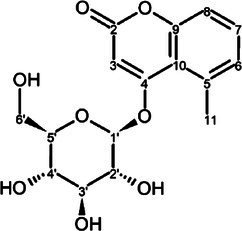	8.0 min (5 mM ammonium formate)	339.1070 (C_16_H_19_O_8_, 339.1079)	337.0927 (C_16_H_17_O_8_, 337.0923)	C_16_H_18_O_8_	2	–	^b^
					3	6.03 (s)	93.3
					4	–	169.2^c^
					5	–	138.7^c^
					6	7.24 (d, 7.6)	129.4
					7	7.65 (t, 7.8)	133.7
					8	7.30 (d, 8.1)	116.1
					9	–	155.7^c^
					10	–	114.7^c^
					11	2.77 (s)	23.7
					1′	5.34 (d, 7.8)	100.3
					2′	3.73 (dd, 9.2, 7.8)	73.9
					3′	3.62 (t, 9.2)	77.1
					4′	3.52 (m)	70.1
					5′	3.68 (ddd, 10, 5.6, 2.3)	77.9
					6′	3.78 (dd, 12.4, 5.6) 3.94 (dd, 12.4, 2.3)	61.5

*Coupling constants (J in Hz) in parentheses*.

*s, singlet; d, doublet; t, triplet; m, multiplet; dd, doublet of doublets; dt, doublet of triplets*.

a *fragment ions are reported in* Supplementary Figures S5, S10, S11, S16.

b *not observed*.

c *observed in HMBC*.

**Figure 3:**
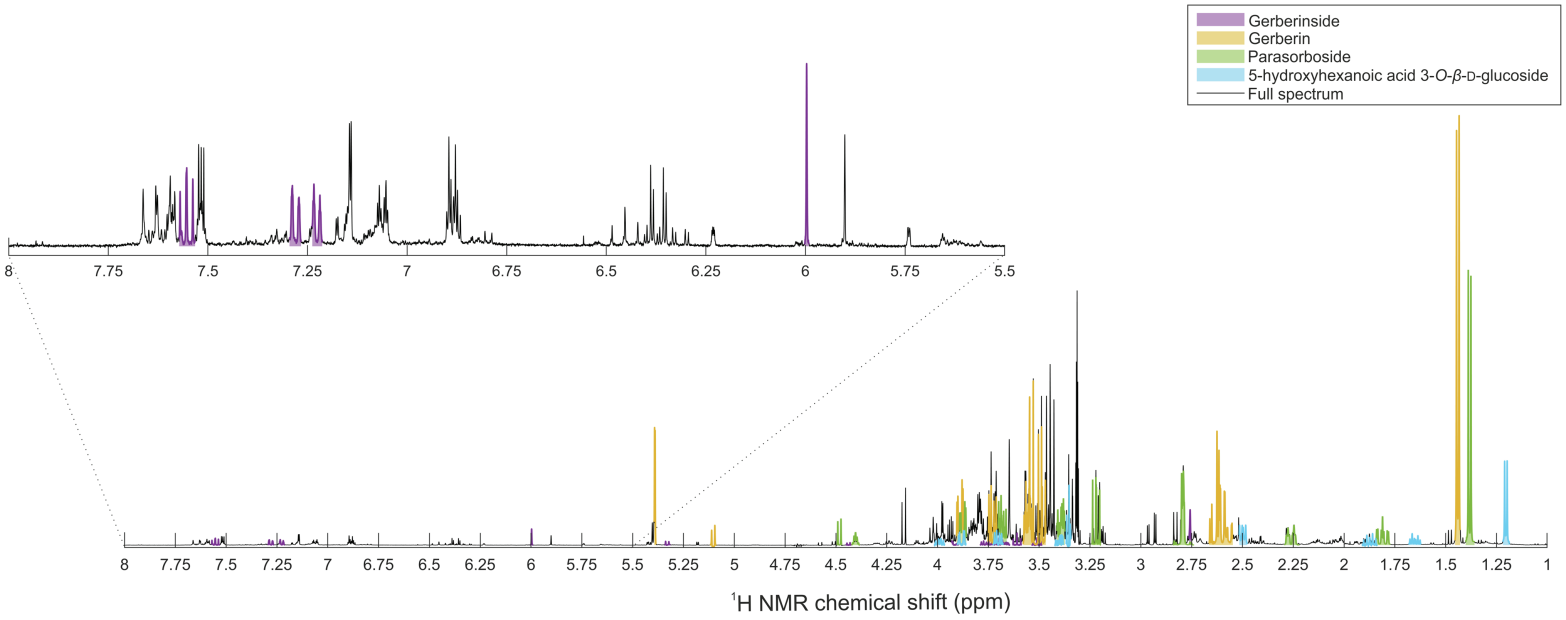
Representative annotated ^1^H NMR spectrum of young leaves of the powdery-mildew-resistant variety Snowking extracted in MeOD-D_2_O (1:1, v/v).

To the best of our knowledge, the spectral data for 5-hydroxyhexanoic acid 3-*O*-*β*-D-glucoside do not correspond to data of any compound previously published, but they agree partially with the data for methyl and ethyl esters previously described (Tschesche et al., 1971; Numata et al., 1990), who considered them to be artefacts. The 5-hydroxyhexanoic acid 3-*O*-*β*-D-glucoside (purity 95%) was isolated as a colorless amorphous substance. Gerberin and parasorboside were isolated and identified as an inseparable mixture (Yrjönen et al., 2002). The NMR data obtained were in good agreement with those published (Nagumo et al., 1989), as well for gerberinside (Nagumo et al., 1989; He et al., 2014) although different solvents were used and H-3′ and H-5′ of the structure were probably erroneously assigned (He et al., 2014).

### Quantitative ^1^H NMR of the Isolated Compounds

The concentrations of 5-hydroxyhexanoic acid 3-*O*-*β*-D-glucoside, gerberin, parasorboside, and gerberinside differed for young and old leaves in resistant and susceptible varieties of *Gerbera* (Supplementary Figure S17; Supplementary Table S1). Independent of leaf age, leaves of resistant varieties generally accumulated more parasorboside (*p* ≤ 0.001) and gerberinside (*p* ≤ 0.001), with younger leaves containing higher concentrations of parasorboside (*p* = 0.01) and gerberinside (*p* = 0.002) than older leaves. This was also the case for susceptible varieties with younger leaves containing more parasorboside (*p* ≤ 0.001) and gerberinside (*p* ≤ 0.001) than older leaves. Greater amounts of gerberin occurred only in the leaves of resistant standard gerbera (*p* ≤ 0.001), but not in the resistant mini varieties.

### Resistance Detection Model

**Figure 4:**
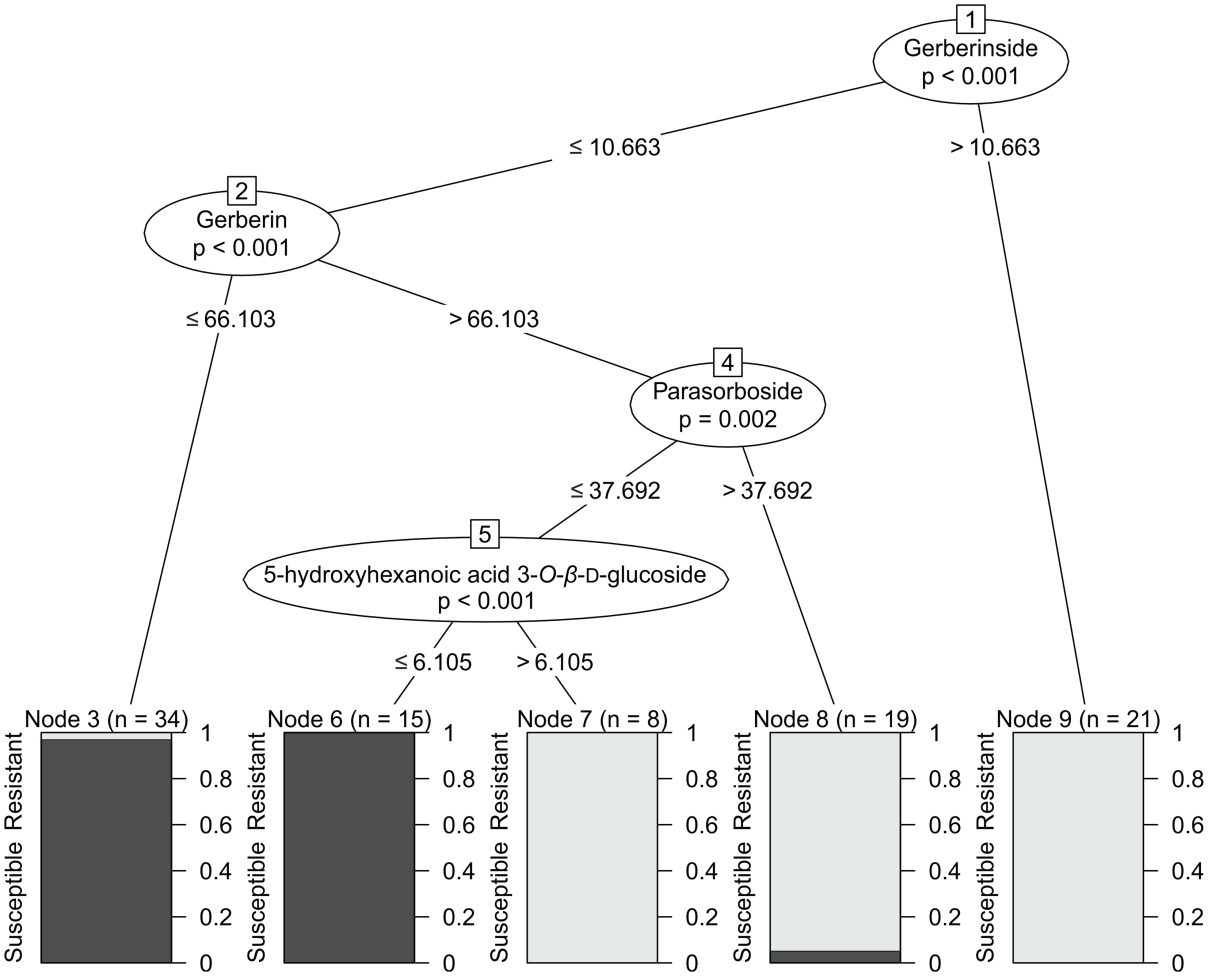
Decision tree showing the concentrations of the selected metabolites responsible for resistance to powdery mildew based on the ^1^H NMR spectra of gerbera leaves extracted in MeOD-D_2_O (1:1, v/v). Concentrations are expressed as mg/g of dry leaves. Nodes are either numbered in squares or as Node #. Bars show proportions of plants described in the training subset as resistant (light color) and susceptible (dark color).

Based on the selected candidate markers for resistance, a decision tree predictive model was constructed to predict the detection of resistance for breeding purposes. Decision trees are one of the most popular classification algorithms in current use in data mining and machine learning. The algorithms can easily handle multi-class discrimination and its constructed decision tree has a structure that is easy to understand as a flowchart. The algorithms are based on a recursive procedure which logically combines a sequence of simple tests (Kotsiantis, 2013). Each sample was characterized only by the concentrations of the candidate markers. To reduce the risk of over-fitting, 30% of the dataset was not used to construct the model but rather to evaluate the model. The resulting predictive model achieved 92.5% accuracy, 90% sensitivity, and 95% specificity. It is depicted in Figure 4. Gerberinside was the key compound for class discrimination, with varieties rich in gerberinside being mildew-resistant. When the concentration of gerberinside is lower, greater concentrations of gerberin and parasorboside lead to resistance to powdery mildew. If a smaller amount of parasorboside was present, then the resistance was dependent on the concentration of 5-hydroxyhexanoic acid 3-*O*-*β*-D-glucoside. A total of 52 samples were used as an external validation dataset to test the applicability of the model for breeding purposes. The model showed an accuracy of 57.7%, sensitivity of 72%, and specificity of 44.44% (Table 2). Four varieties already used in the first test set were included in the validation set. They were predicted within the same class. The same varieties but derived from different breeders have also been predicted within the same class.

**Table 2:** Decision tree confusion matrix classification of gerbera leaves resistant and susceptible to mildew in the validation sample set extracted in MeOD-D_2_O (1:1, v/v).

		Predicted class	
		Resistant	Susceptible
Real class	Resistant	18	7
	Susceptible	15	12

*Misclassification error: 44%, sensitivity: 72%, specificity: 44%*.

## Discussion

Discriminating analysis of resistant and susceptible gerbera plants showed that gerberin, parasorboside, gerberinside, and 5-hydroxyhexanoic acid 3-*O*-*β*-D-glucoside can be used as biomarkers for resistance to powdery mildew. Independent of leaf age, these compounds showed greater concentrations in the resistant compared to the susceptible varieties. These polyketide-derived glycosylated molecules are compounds well-known present in gerbera (Bohlmann et al., 1973; Teeri et al., 2006), where the aglycones are considered to play a role in defending the plant against pathogens (Koskela et al., 2001, 2011; Pietiäinen et al., 2016). Gerberin and parasorboside are produced in large amounts in all gerbera tissues and are described as bitter tasting glycosidic lactones (Yrjönen et al., 2002; Pietiäinen et al., 2016). Gerberin aglycone is known as 5,6-dihydro-4-hydroxy-6-methyl-2*H*-pyran-2-one. Gerbera and its close relatives produce rare coumarin-like compounds, such as 4-hydroxy-5-methylcoumarin, which is the aglycone component of gerberinside. This compound is somewhat taxonomically restricted to *Gerbera* spp. (Inoue et al., 1989; Nagumo et al., 1989; Pietiäinen et al., 2016), however, its derivatives occur in other taxa within the Asteraceae family, such as in *Mutisia* spp. (Zdero et al., 1988). Moreover, an occurrence in taxonomically unrelated *Diospyros* spp. has been reported (Paknikar et al., 1996). It is important to mention, that this compound is formed by a polyketide synthase in the acetate-mevalonate pathway and not the shikimate (phenylpropanoid) pathway, the source of relatively common coumarins found particularly in plants of the Apiaceae and Rutaceae families (Bourgaud et al., 2006; Pietiäinen et al., 2016).

Gerbera expresses three genes encoding for 2-pyrone synthases (*G2PS1*, *G2PS2*, and *G2PS3*) which are chalcone synthase-like polyketide synthases with altered starter substrate specificity (Helariutta et al., 1996). *G2PS1* is present in the whole plant and is responsible for the synthesis of 4-hydroxy-6-methyl-2-pyrone (triacetolactone), a putative precursor of gerberin and parasorboside (Eckermann et al., 1998). *G2PS2*, expressed in the leaf blades and inflorescences, and *G2PS3*, expressed in the roots, are necessary for the biosynthesis of 4-hydroxy-5-methylcoumarin. *G2PS2* and *G2PS3* lead to the formation of 4,7-dihydroxy-5-methylcoumarin.

Chemometrics gives us a tool to study the relationships among multiple metabolites at the same time. The decision tree explains more complex relationships between compounds, which are not elucidated by a univariate statistical approach. The main node of the decision tree is gerberinside, which suggests that this compound plays the most important role in defining resistance. A comparable result was obtained from the OPLS-DA. Signals from the compound were assigned the highest values of absolute *p*(corr) and significance by the *t*-test. The antifungal activity of plant coumarins has been investigated and confirmed (Sardari et al., 2000). In particular, gerberinside aglycone (4-hydroxy-5-methylcoumarin) was observed to be more effective than gerberin aglycone, triacetolactone, and sorbic acid for inhibiting the growth of *Botrytis cinerea*, *Heterobasidium annosum*, and *Rhizoctonia solani* in *in vitro* tests using artificial media (Koskela et al., 2011). According to the decision tree, resistant varieties with low concentrations of gerberinside were characterized by greater concentrations of gerberin and parasorboside. Parasorboside is regarded as a precursor of parasorbic acid, the plant form of the widely used food preservative sorbic acid (Tschesche et al., 1971; Koskela et al., 2011). In agreement with our results, the concentrations of gerberin and parasorboside varied greatly between different gerbera varieties, with varieties resistant to *B. cinerea* containing greater concentrations than the more susceptible (Koskela et al., 2001). In most varieties, gerberin was most abundant in the flowers, whereas the highest level of parasorboside was observed in both the leaf and the scape (Koskela et al., 2001). Plants in which the pathway was inhibited with antisense *G2PS1* blocking the synthesis of gerberin and parasorboside showed increased susceptibility to *B. cinerea* (Koskela et al., 2011). Gerberin aglycone inhibited the growth of *B. cinerea*, *H. annosum*, and *R. solani* (Koskela et al., 2011). In addition, parasorboside has been demonstrated to be an antifeedant agent for the larvae of the yellow butterfly *Eurema hecabe mandarina* (Numata et al., 1990). Interestingly, the lines with downregulated *G2PS1* (Koskela et al., 2011) also showed lower abundance of the 4-hydroxy-5-methylcoumarin. This compound was far more active than gerberine aglycone in disc diffusion antifungal assay. They speculated that the biosynthesis of 4-hydroxy-5-methylcoumarin is a further activity of *G2PS1*, or that there are other similar enzymes in the plant that get co-downregulated in the antisense plants. Later the same group found that indeed there is a pair of related enzymes, *G2PS2* and *G2PS3*, and that these are pentaketide synthases involved in the synthesis of 4,7-dihydroxy-5-methylcoumarin (Pietiäinen et al., 2016). The further pathway is unknown, but a reductase enzyme is likely required to complete the pathway for the biosynthesis of 4-hydroxy-5-methylcoumarin (Pietiäinen et al., 2016).

The decision tree suggested that 5-hydroxyhexanoic acid 3-*O*-*β*-D-glucoside may also play a role in resistance to mildew. Strong structural similarities to parasorboside suggest that this compound may be part of the gerberin pathway. Further investigation is needed to clarify the role of this compound in gerbera.

The segregated class of isolated compounds was glycosylated. As the first chemical barriers to attacking organisms, preformed inhibitory compounds play a key role in the early stages of plant defense. However, these compounds may also be toxic for plant itself. One of the strategies implemented by the plants, and shaped by continuous evolutionary forces regulated by the adaptation of the host plant, is therefore conjugation of the toxic defense compound with various organic molecules, including sugars. The detoxification of a harmful compound by glycosylation can generate a non-toxic agent and often leads to sequestration in a storage compartment, such as the cell vacuole (Jones and Vogt, 2001; Gachon et al., 2005). When a pathogen attacks, the detoxified compound is activated by glycosyl hydrolases released by the host plant or the invading organism (Osbourn, 1996; Minic, 2008; Murphy et al., 2011). Glycosylation increases the water solubility and also stability of hydrophobic metabolites, improving their biodistribution and metabolism (Jones and Vogt, 2001; Gachon et al., 2005).

NMR-based metabolomics coupled with chemometrics can detect plant metabolites involved in resistance to powdery mildew in plants. Based on the contents of gerberin, parasorboside, gerberinside, and 5-hydroxyhexanoic acid 3-*O*-*β*-D-glucoside, model for the prediction of resistance to a powdery mildew was developed. Collecting more data on more varieties and on more time points during crop growth will improve the model even further. Such a model enables screening of existing varieties and also breeding population intended for the development of new varieties. As such, the metabolites can function as biomarkers for resistance to mildew and represent a crucial tool for resistance breeding programs. Especially so since powdery mildew is an obligate biotroph pathogen, which until now only could be tested by using relatively small scale, labor and capital intensive plant bioassays; validation of the single compounds by *in vitro* bioassays is hampered. Single compounds would need to be separated, whereby the separation of gerberin and parasorboside would present a major challenge (Yrjönen et al., 2002). Even if compounds would be applied as a mixture, powdery mildew as an obligate fungus does not grow on artificial substrates to which the compounds in question could be added. Here we have an alternative metabolomics-based approach to create decision trees that enabled us to detect the compounds responsible for plant resistance and to determinate their synergies and relative contributions to the overall effect. This approach seems to be particularly important for obligate biotrophic plant pathogens, such as powdery mildew, for which bioassays are not easy to carry out.

## Data Availability Statement

The raw data supporting the conclusions of this article will be made available by the authors, without undue reservation.

## Author Contributions

KL and JH designed the study. JB-M and EO organized and implemented the sampling. AM and JH performed the NMR experiments, analyzed the data, and derived the model. AM wrote the manuscript in consultation with JH and KL. PM carried on M.S. analysis and interpretation. MM contributed on NMR structural elucidation. JT contributed to compound purification. JB-M, EO, PK, KS, PM, MM, and JT reviewed and edited the manuscript. PK and KS provided resources. KL and PK acquired funding. All authors contributed to the article and approved the submitted version.

## Funding

This work was supported by the Dutch Top Sector project “Natural resistance to powdery mildew” (TU 18007). We thank the Dutch Greenhouse Horticulture (Glastuinbouw Nederland), the Foundation Kijk, the Dutch gerbera and rose growers’ association, and the cooperation Royal FloraHolland for their financial assistance. This study was also supported by the European Regional Development Fund-Project No. CZ.02.1.01/0.0/0.0/16_019/0000845, by the SGS CZU FAPPZ (Grant No: SV21-12-21310) and by a METROFOOD-CZ research infrastructure project (MEYS Grant No: LM2018100) including access to its facilities.

## Conflict of Interest

The authors declare that the research was conducted in the absence of any commercial or financial relationships that could be construed as a potential conflict of interest.

## Publisher’s Note

All claims expressed in this article are solely those of the authors and do not necessarily represent those of their affiliated organizations, or those of the publisher, the editors and the reviewers. Any product that may be evaluated in this article, or claim that may be made by its manufacturer, is not guaranteed or endorsed by the publisher.
