# Supplementary material for: Polyketide Derivatives in the Resistance of Gerbera hybrida to Powdery Mildew
Source: Front Plant Sci. 2022 Jan 6;12:790907. doi: 10.3389/fpls.2021.790907 (PMC8770985; doi:10.3389/fpls.2021.790907)
Supplement: Supplementary file 1 [file Data_Sheet_1.docx]

Supplementary Material

# Supplementary Tables

**Supplementary Table S1.** Pairwise analysis of 5-hydroxyhexanoic acid 3-*O*-*β*-D-glucoside, gerberin, parasorboside, and gerberinside of young and old leaves of varieties resistant and susceptible to powdery mildew extracted in MeOD-D_2_O (1:1, v/v).

|  | 5-hydroxyhexanoic acid 3-*O*-*β*-D-glucoside | | | Gerberin | | | Parasorboside | | | Gerberinside | | |
| --- | --- | --- | --- | --- | --- | --- | --- | --- | --- | --- | --- | --- |
| Class | $\bar{x}$* | ±SD | p-value | $\bar{x}$* | ±SD | p-value | $\bar{x}$* | ±SD | p-value | $\bar{x}$* | ±SD | p-value |
| Resistant^a^ | 12.58 | 9.09 | 0.63 | 73.72 | 47.47 | 0.31 | 39.69 | 12.62 | 8.60  e-04 | 11.36 | 5.46 | 6.05  e-08 |
| Susceptible^a^ | 11.71 | 10.16 |  | 65.84 | 36.61 |  | 32.56 | 10.07 |  | 6.88 | 2.44 |  |
| Young Resistant^b^ | 13.78 | 10.58 | 0.95 | 74.16 | 43.78 | 0.69 | 43.65 | 11.95 | 0.02 | 13.48 | 6.03 | 5.80  e-05 |
| Young Susceptible^b^ | 13.99 | 12.54 |  | 69.89 | 39.26 |  | 37.56 | 7.82 |  | 8.46 | 1.95 |  |
| Old  Resistant^b^ | 11.38 | 7.30 | 0.28 | 73.29 | 51.65 | 0.31 | 35.72 | 12.20 | 0.01 | 9.23 | 3.89 | 5.16  e-06 |
| Old Susceptible^b^ | 9.45 | 6.51 |  | 61.81 | 33.93 |  | 27.56 | 9.67 |  | 5.31 | 1.79 |  |
| Resistant young^b^ | 13.78 | 10.58 | 0.31 | 74.16 | 43.78 | 0.94 | 43.65 | 11.95 | 0.01 | 13.48 | 6.03 | 1.96  e-03 |
| Resistant  old^b^ | 11.38 | 7.30 |  | 73.29 | 51.65 |  | 35.72 | 12.20 |  | 9.23 | 3.89 |  |
| Susceptible young^b^ | 13.99 | 12.54 | 0.08 | 69.89 | 39.26 | 0.40 | 37.56 | 7.82 | 4.63  e-05 | 8.46 | 1.95 | 1.93  e-08 |
| Susceptible old^b^ | 9.45 | 6.51 |  | 61.81 | 33.93 |  | 27.56 | 9.67 |  | 5.31 | 1.79 |  |
| Mini  resistant^b^ | 12.51 | 9.60 | 0.56 | 34.31 | 25.48 | 0.19 | 35.09 | 9.47 | 0.94 | 14.92 | 5.43 | 3.53  e-09 |
| Mini susceptible^b^ | 11.22 | 7.55 |  | 40.83 | 8.04 |  | 35.32 | 11.99 |  | 7.48 | 2.19 |  |
| Standard resistant^b^ | 12.65 | 8.72 | 0.88 | 113.13 | 26.87 | 0.01 | 44.28 | 13.82 | 3.30  e-06 | 7.79 | 2.22 | 0.02 |
| Standard susceptible^b^ | 12.22 | 12.35 |  | 90.87 | 36.97 |  | 29.80 | 6.83 |  | 6.28 | 2.57 |  |
| Resistant  mini^b^ | 12.51 | 9.60 | 0.95 | 34.31 | 25.48 | 7.79  e-17 | 35.09 | 9.47 | 3.91  e-03 | 14.92 | 5.43 | 1.11  e-08 |
| Resistant standard^b^ | 12.65 | 8.72 |  | 113.13 | 26.87 |  | 44.28 | 13.82 |  | 7.79 | 2.22 |  |
| Susceptible mini^b^ | 11.22 | 7.55 | 0.70 | 40.83 | 8.04 | 1.13  e-09 | 35.32 | 11.99 | 0.03 | 7.48 | 2.19 | 0.06 |
| Susceptible standard^b^ | 12.22 | 12.35 |  | 90.87 | 36.97 |  | 29.80 | 6.83 |  | 6.28 | 2.57 |  |

*average concentration expressed as mg/g dry leaves. ^a^N=60, ^b^N=30

**Supplementary Table S2** Description of the independent validation dataset.

| Variety | Type | Resistance definition according to the breeder | Location |
| --- | --- | --- | --- |
| Clasico | Standard | Susceptible | Breeder D |
| Cream Beach | Standard | Susceptible | Breeder D |
| Dark Diamond | Standard | Resistant | Breeder D |
| Don Leo | Standard | Resistant | Breeder D |
| Evi | Standard | Susceptible | Breeder D |
| Full moon | Standard | Resistant | Breeder D |
| Mandarina | Standard | Susceptible | Breeder D |
| Peptalk | Standard | Susceptible | Breeder D |
| Mango | Standard | Susceptible | Breeder D |
| Red Explosion | Standard | Resistant | Breeder D |
| Rodeo | Standard | Resistant | Breeder D |
| Romance | Standard | Susceptible | Breeder D |
| Submarine | Standard | Resistant | Breeder D |
| White House | Standard | Susceptible | Breeder D |
| Albino | Mini | Resistant | Breeder D |
| Allure | Mini | Susceptible | Breeder D |
| Banana | Mini | Susceptible | Breeder D |
| Cafe | Mini | Resistant | Breeder D |
| Cassis | Mini | Resistant | Breeder D |
| Delmonte | Mini | Resistant | Breeder D |
| Noud | Mini | Susceptible | Breeder D |
| Dorito | Mini | Susceptible | Breeder D |
| Kimsey | Mini | Resistant | Breeder D |
| Suri | Mini | Susceptible | Breeder D |
| Navelino | Mini | Resistant | Breeder D |
| Delphi | Mini | Resistant | Breeder A |
| Kimsey | Standard | Susceptible | Breeder A |
| Jumbo | Mini | Resistant | Breeder A |
| Mokka | Mini | Susceptible | Breeder A |
| Passoa | Standard | Resistant | Breeder A |
| Madeira | Standard | Susceptible | Breeder A |
| Flamenco | Standard | Susceptible | Breeder A |
| Contigo | Standard | Susceptible | Breeder A |
| Betty Boop | Mini | Susceptible | Breeder A |
| Lido | Mini | Susceptible | Breeder A |
| Cafe del mar | Standard | Resistant | Breeder A |
| Monza | Mini | Resistant | Breeder A |
| Bravia | Mini | Resistant | Breeder A |
| Petticoat | Mini | Resistant | Breeder A |
| Franky | Mini | Resistant | Breeder A |
| Suri | Standard | Susceptible | Breeder E |
| Cafe | Standard | Resistant | Breeder E |
| Prosseco | Mini | Susceptible | Breeder E |
| Caramba | Standard | Resistant | Breeder E |
| Nirvana | Mini | Resistant | Breeder E |
| Maserati | Mini | Susceptible | Breeder E |
| Dixon | Standard | Resistant | Breeder E |
| Caprise | Standard | Susceptible | Breeder E |
| Huski | Standard | Susceptible | Breeder E |
| Waakita | Standard | Resistant | Breeder E |
| Kimsey | Standard | Susceptible | Breeder E |
| Kimsey | Standard | Susceptible | Breeder F |

**Supplementary Table S3.** Description of the acquisition of the 2D NMR experiments.

| 2D experiment | Description |
| --- | --- |
| DQF-COSY | A phase-sensitive 2D correlation spectroscopy (DQF-COSY) with Double Quantum Filter and presaturation (Bruker pulse sequence *cosydfphpr*) was obtained with 1.0 s relaxation delay, 6510 Hz spectral width in both dimensions (1H chemical shift axes). Twenty-four scans were acquired per 521 increments for an overall experimental time of 5hr and 56 min. Zero filled data to 4,096 × 4,096 points and a sine2 bell-shaped window function shifted by /2 in the F1 and /4 in the F2 dimension was applied. Automatically phased and calibrated to the internal standard (TSP = 0.0 p.p.m.). |
| HMBC | A phase-sensitive ge-2D heteronuclear multiple bond correlation (HMBC) (Bruker pulse sequence *hmbcetgpl3nd*) was acquired using 512 and 4,096 complex points in F1 and F2, respectively. Spectral widths of 6510 Hz in F2 (^1^H chemical shift axis) and 27675 Hz in F1 (^13^C chemical shift axis) yielding t1 and t2 acquisition times of 4 and 314 ms, respectively. 240 scans were acquired for an overall experimental time of 23 hr and 51 min. A sine2 bell-shaped window function shifted by /2 in the F1 and /6 in the F2 dimension was applied. The spectrum was calibrated according to the internal standard (^1^H: TSP = 0 ppm; ^13^C: ethanol = 16.7 ppm). |
| HSQC | A phase-sensitive ge-2D heteronuclear single quantum coherence spectroscopy (HSQC) (Bruker pulse sequence *hsqcgpph*) was acquired using 256 and 1,024 complex points in F1 and F2, respectively. Spectral widths of 6510 Hz in F2 (^1^H chemical shift axis) and 27675 Hz in F1 (^13^C chemical shift axis) yielding t1 and t2 acquisition times of 4 and 314 ms, respectively. 240 scans were acquired for an overall experimental time of 1d 3 hr and 13 min. A sine2 bell-shaped window function shifted by /2 in the F1 and /6 in the F2 dimension was applied. The spectrum was calibrated according to the internal standard (^1^H: TSP = 0 ppm; ^13^C: ethanol = 49.0 ppm). |

**Supplementary Table S4.** Parameters of the liquid chromatography separation and the mass spectrometer settings.

|  | Parameter | Settings |
| --- | --- | --- |
| Liquid chromatography separations | Column | Acclaim™ RSLC 120 C18 column (2.2 μm, 120Å 2.1 × 100 mm; Thermo Fisher Scientific, Waltham, MA, USA), coupled with an Acquity UPLC BEH C18 VanGuard pre-column (Waters, Milford, MA, USA) |
|  | Temperature | 35℃ |
|  | Mobile phase | 5 mM ammonium formate or 0.2% formic acid in water (phase A) and methanol (phase B) |
|  | Gradient elution program | 5% B isocratically for 1.5 min, then from 5% to 70% B over the next 8.5 min, from 70% to 100% B during the next 3 min, held constant for 4 minutes, and then returned to the initial conditions |
|  | Flow rate | 0.25 mL/min |
|  | Injection volume | 5 µL |
| Mass spectrometer | Ion source settings: | End Plate Offset: 500 V  Capillary voltage: 3500 V  Nebulizer gas: 2.0 Bar  Drying gas (N_2_) 8.0 L/min  Drying temperature: 250°C |
|  | Mass Range: | 50-1500 m/z |
|  | Scan rate: | 3x1sec |
|  | Calibration mixture: | Na^+^ Formate clusters |
|  | collision energy | The MS/MS fragmentation spectra collected at three discrete levels of collision energy 20, 40, and 60 eV for each precursor ion |

# Supplementary Figures

**
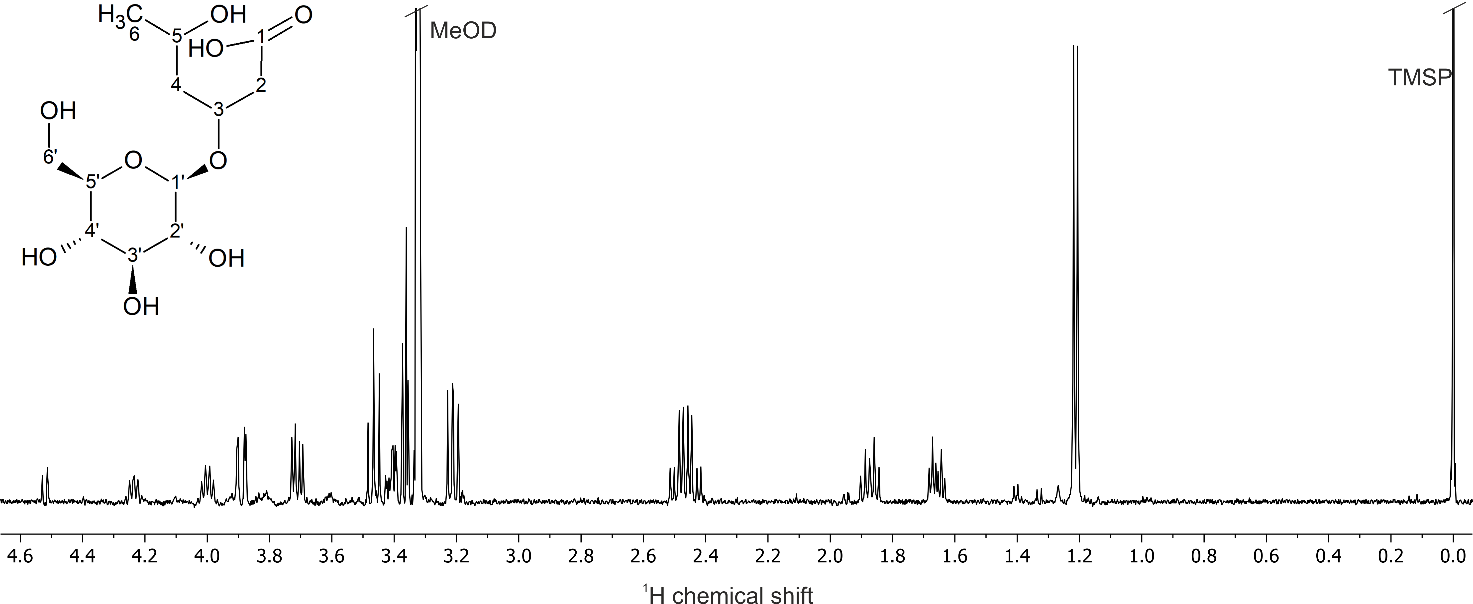
**

**Supplementary Figure S1.** ^1^H NMR spectrum of 5-hydroxyhexanoic acid 3-*O*-*β*-D-glucoside in MeOD-D_2_O (1:1, v/v) (500MHz).

**
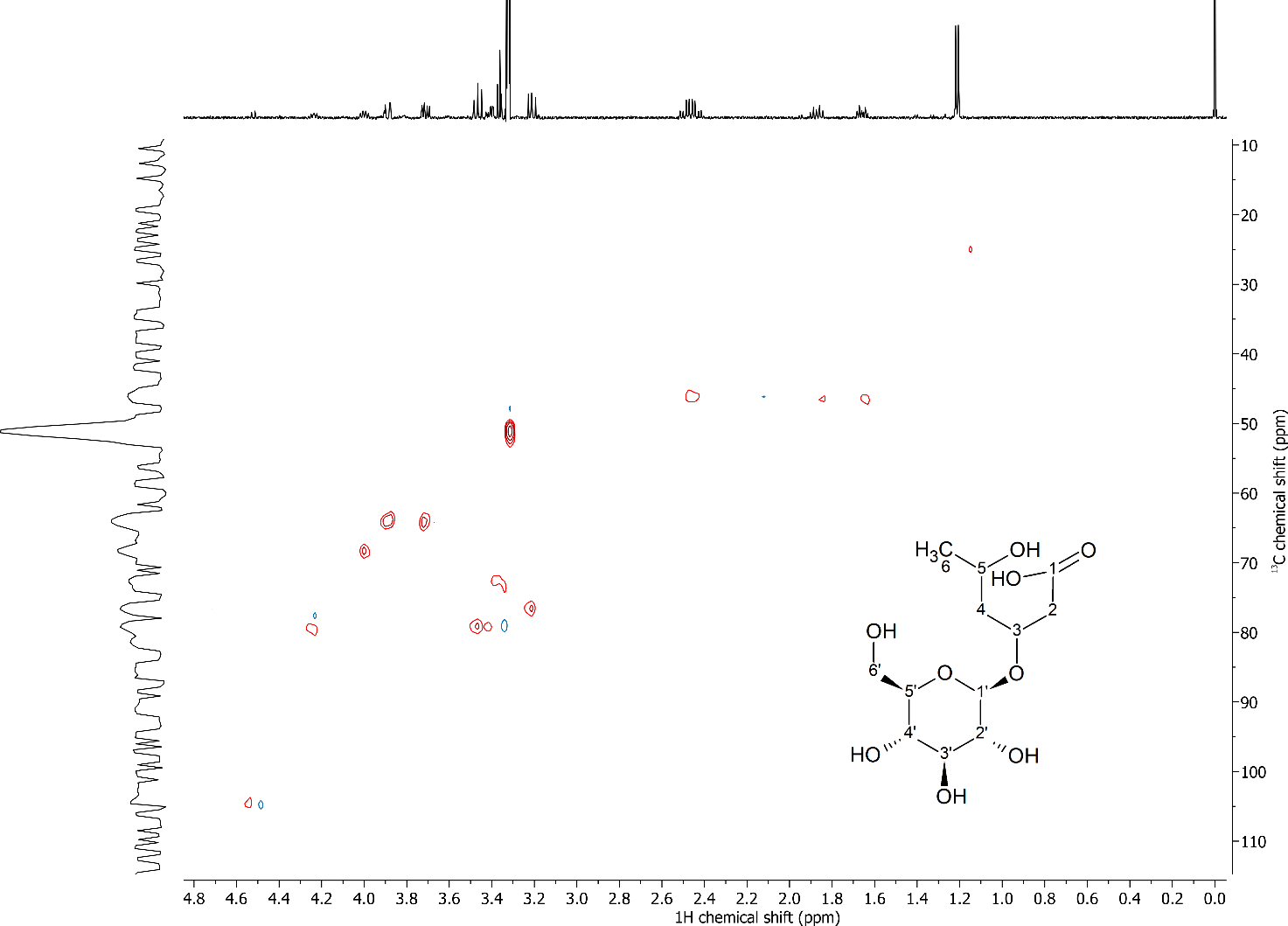
**

**Supplementary Figure S2.** HSQC Spectrum of 5-hydroxyhexanoic acid 3-*O*-*β*-D-glucoside in MeOD-D_2_O (1:1, v/v).

**
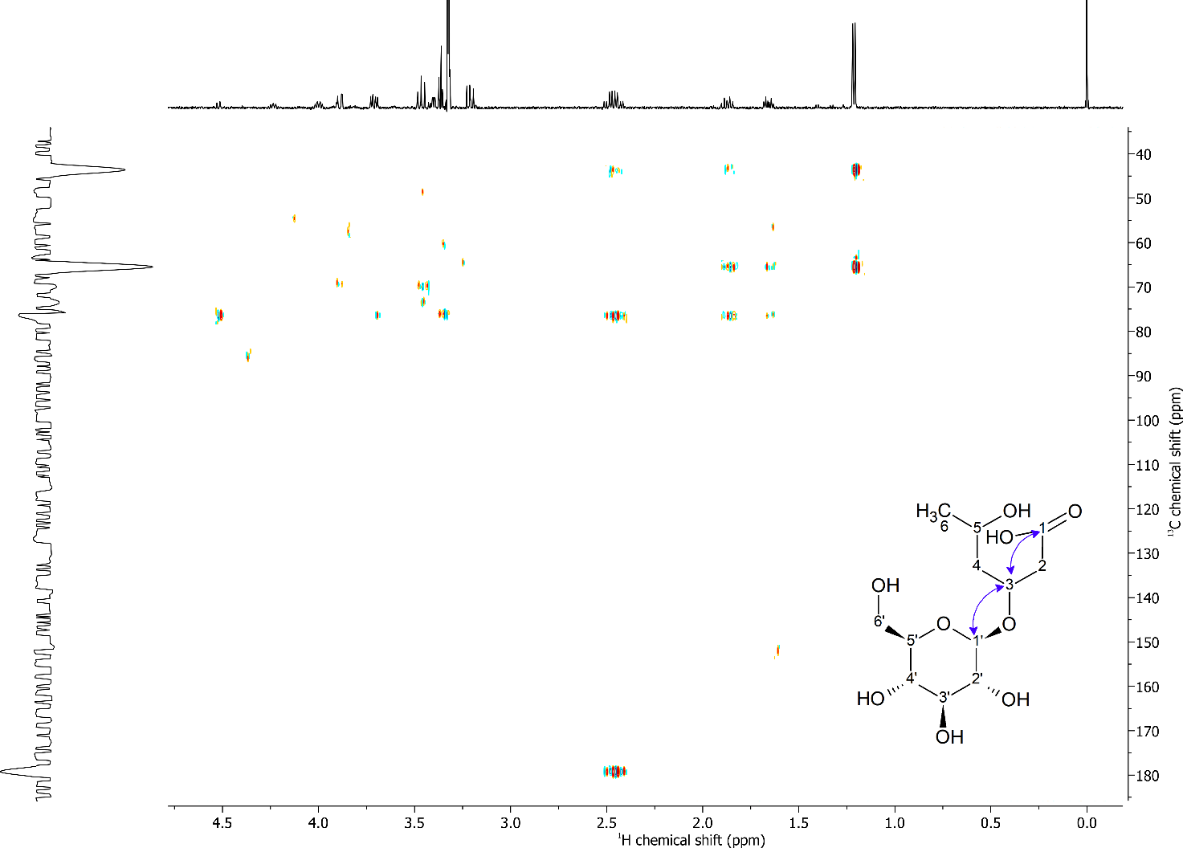
**

**Supplementary Figure S3.** HMBC spectrum and key correlations of 5-hydroxyhexanoic acid 3-*O*-*β*-D-glucoside in MeOD-D_2_O (1:1, v/v).

**
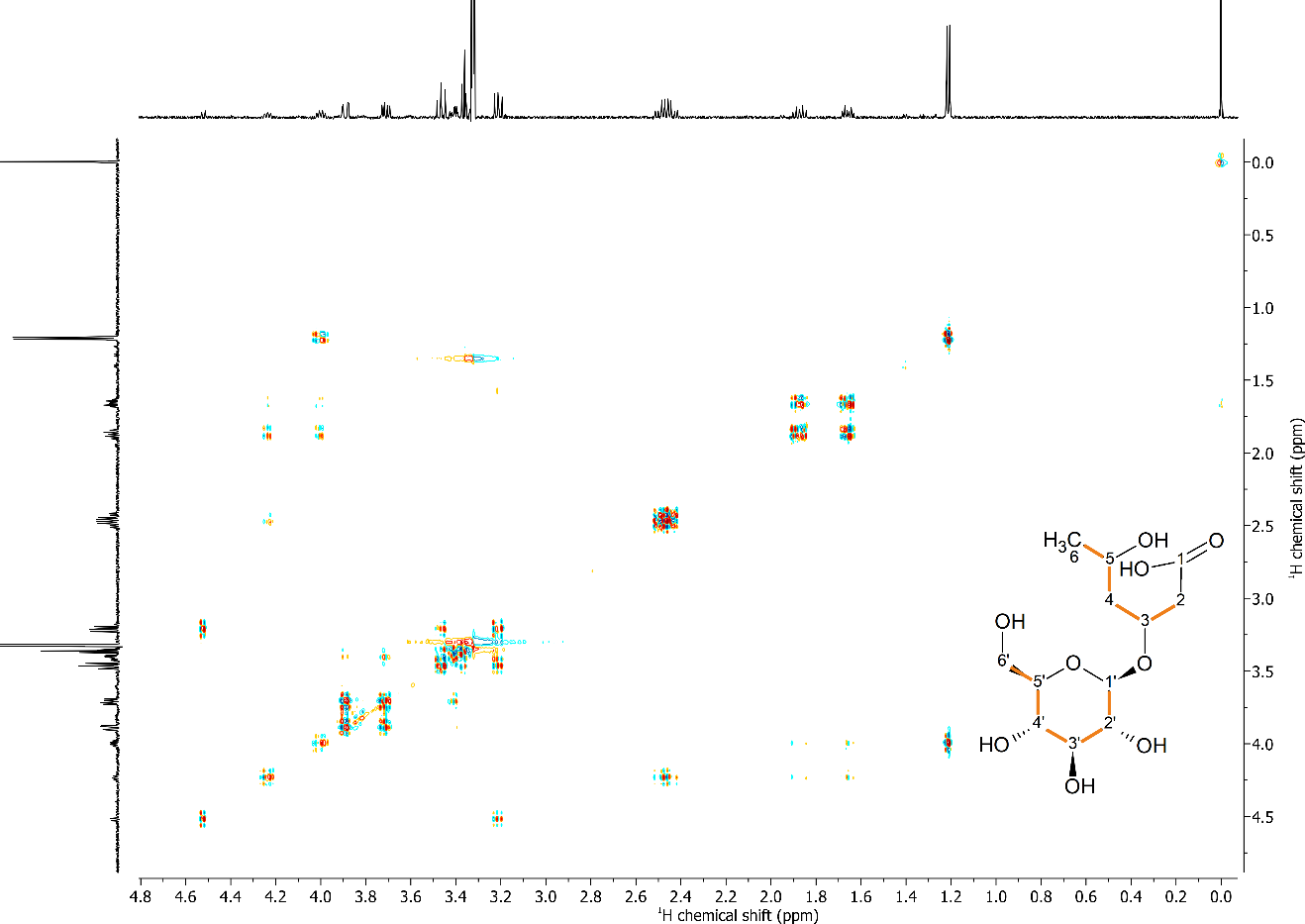
**

**Supplementary Figure S4.** DQF-COSY Spectrum and key correlations of 5-hydroxyhexanoic acid 3-*O*-*β*-D-glucoside in MeOD-D_2_O (1:1, v/v).

**
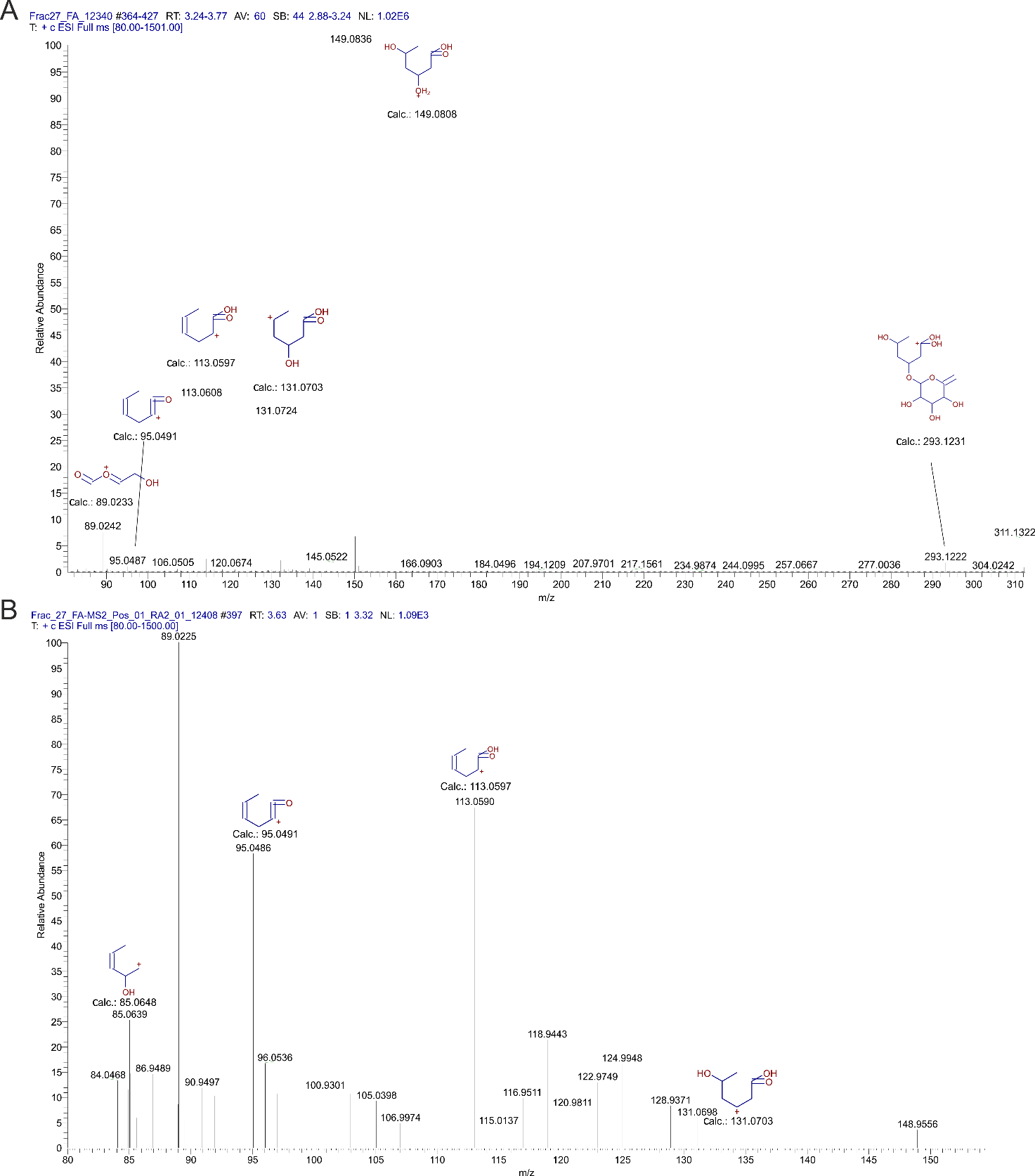
**

**Supplementary Figure S5. (A)** MS spectrum (in-source fragmentation) and proposed fragments of 5-hydroxyhexanoic acid 3-*O*-*β*-D-glucoside at *m/z* 311.1336 [M+H]^+^ and **(B)** MS/MS spectrum of the aglycone at *m/z* 149.0808 [M+H]^+^ (positive mode, 0.2% formic acid and methanol).

**
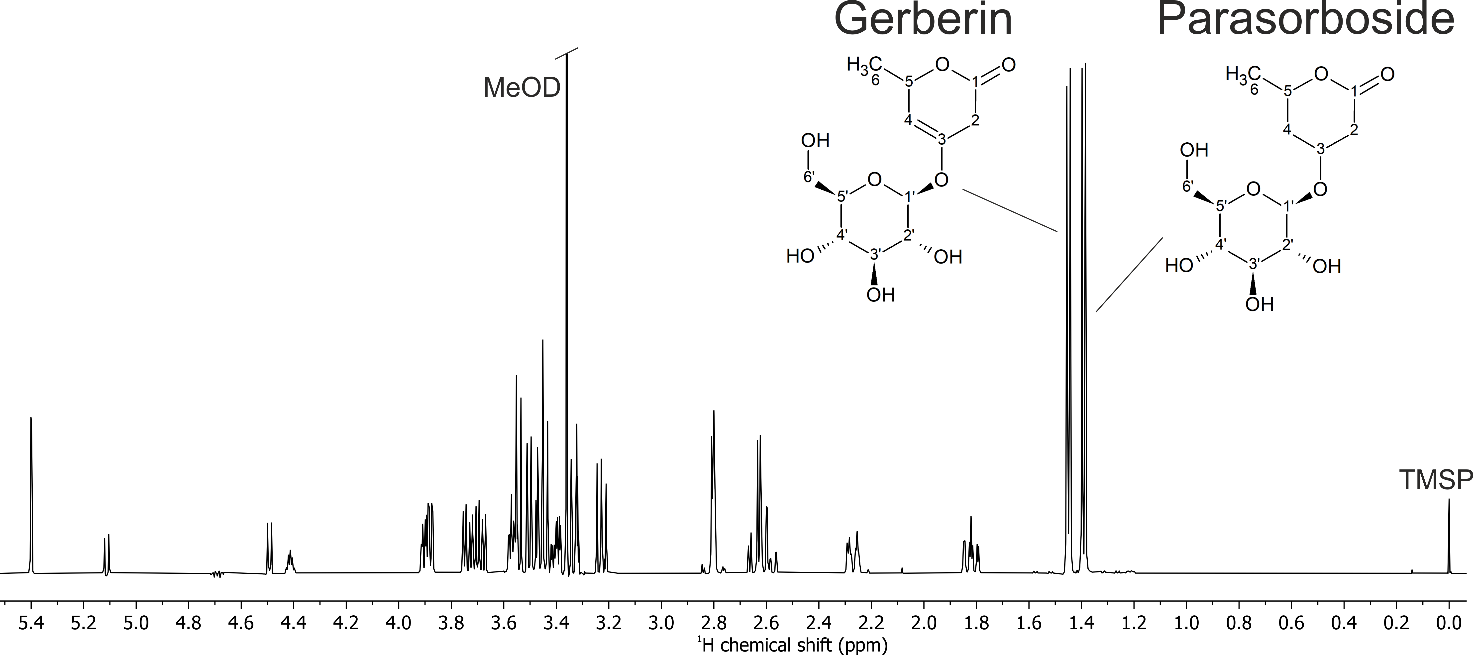
**

**Supplementary Figure S6.** ^1^H NMR spectrum of gerberin and parasorboside in MeOD-D_2_O (1:1, v/v) (500MHz).

**
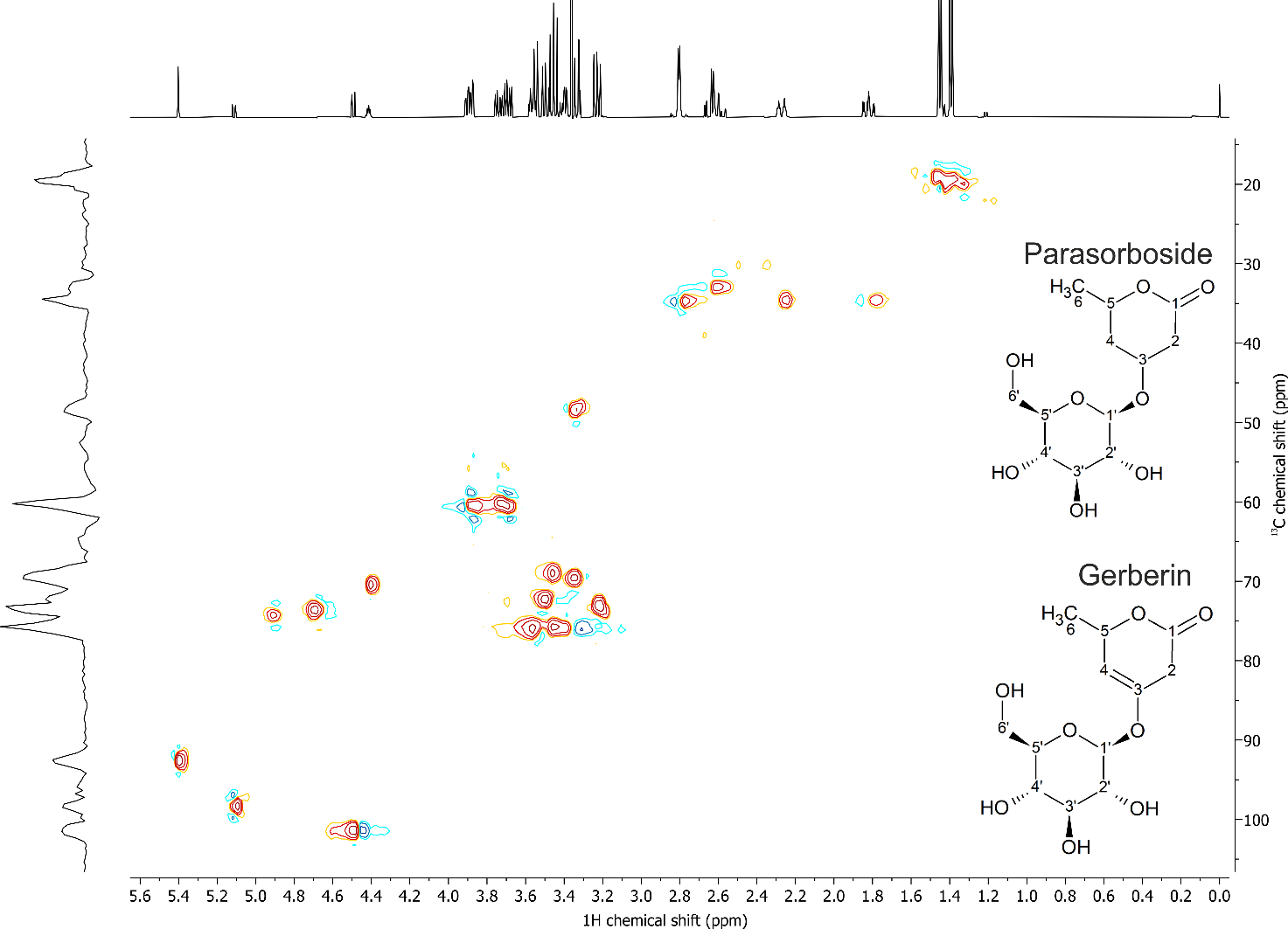
**

**Supplementary Figure S7.** HSQC spectrum of gerberin and parasorboside in MeOD-D_2_O (1:1, v/v).


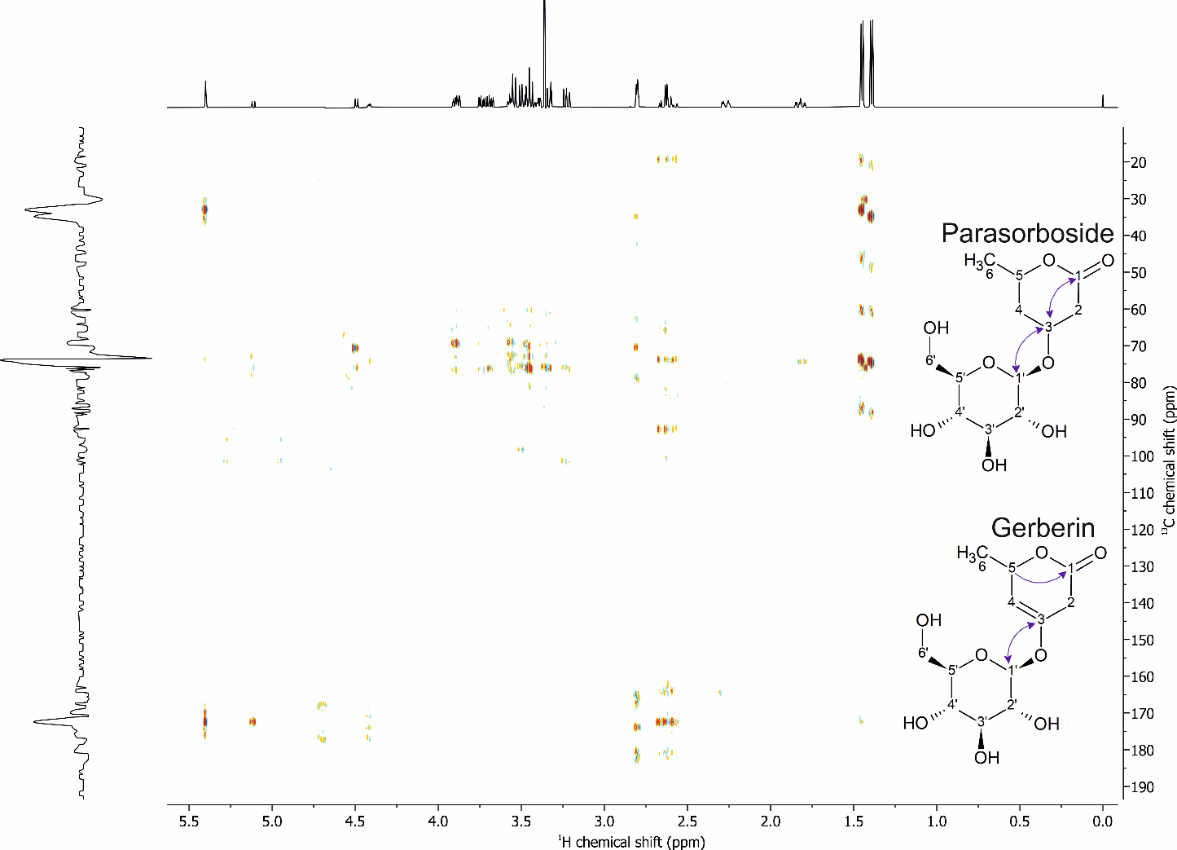


**Supplementary Figure S8.** HMBC spectrum and key correlations of gerberin and parasorboside in MeOD-D_2_O (1:1, v/v).

**
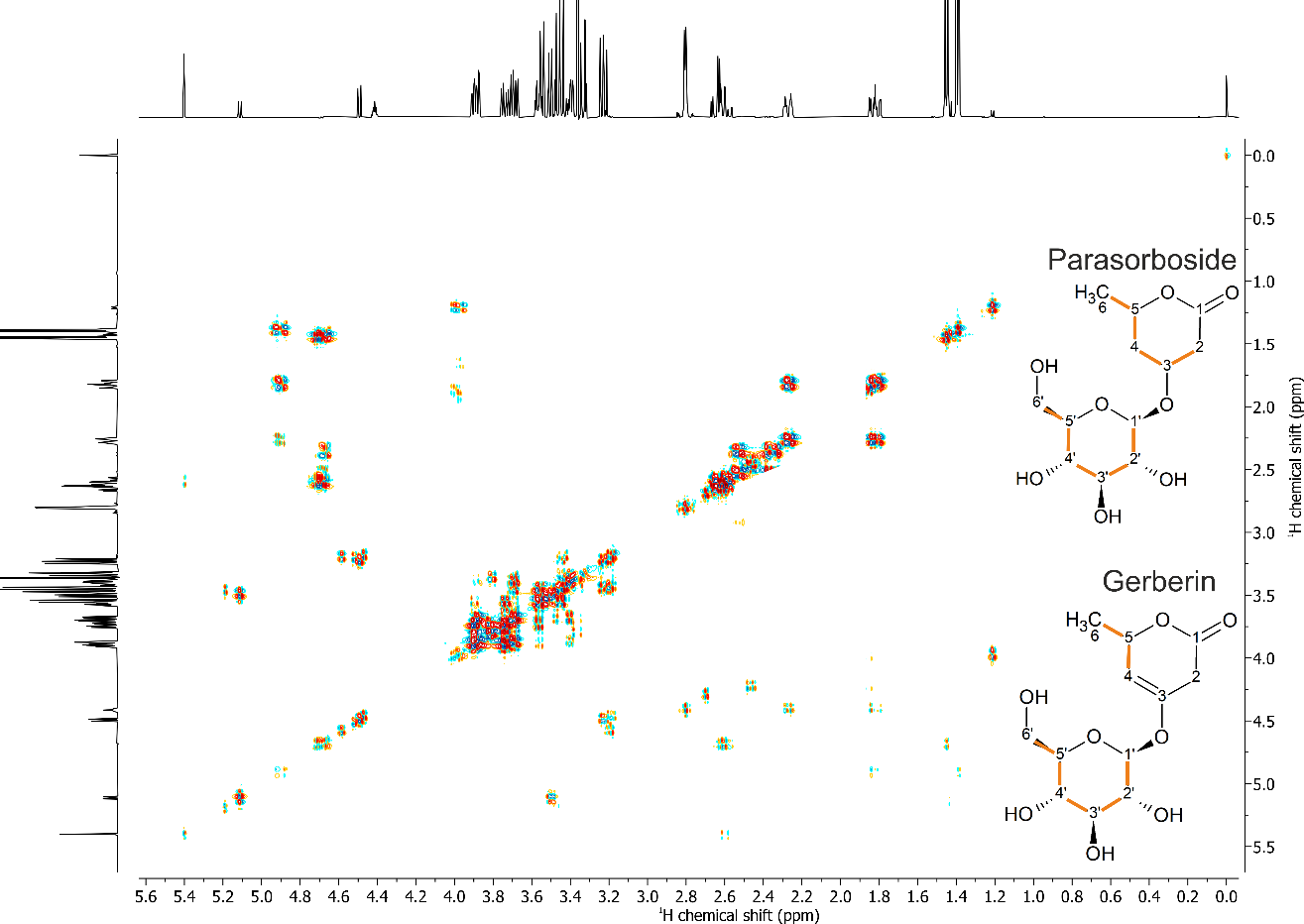
**

**Supplementary Figure S9.** DQF-COSY spectrum and key correlations of gerberin and parasorboside in MeOD-D_2_O (1:1, v/v).

**
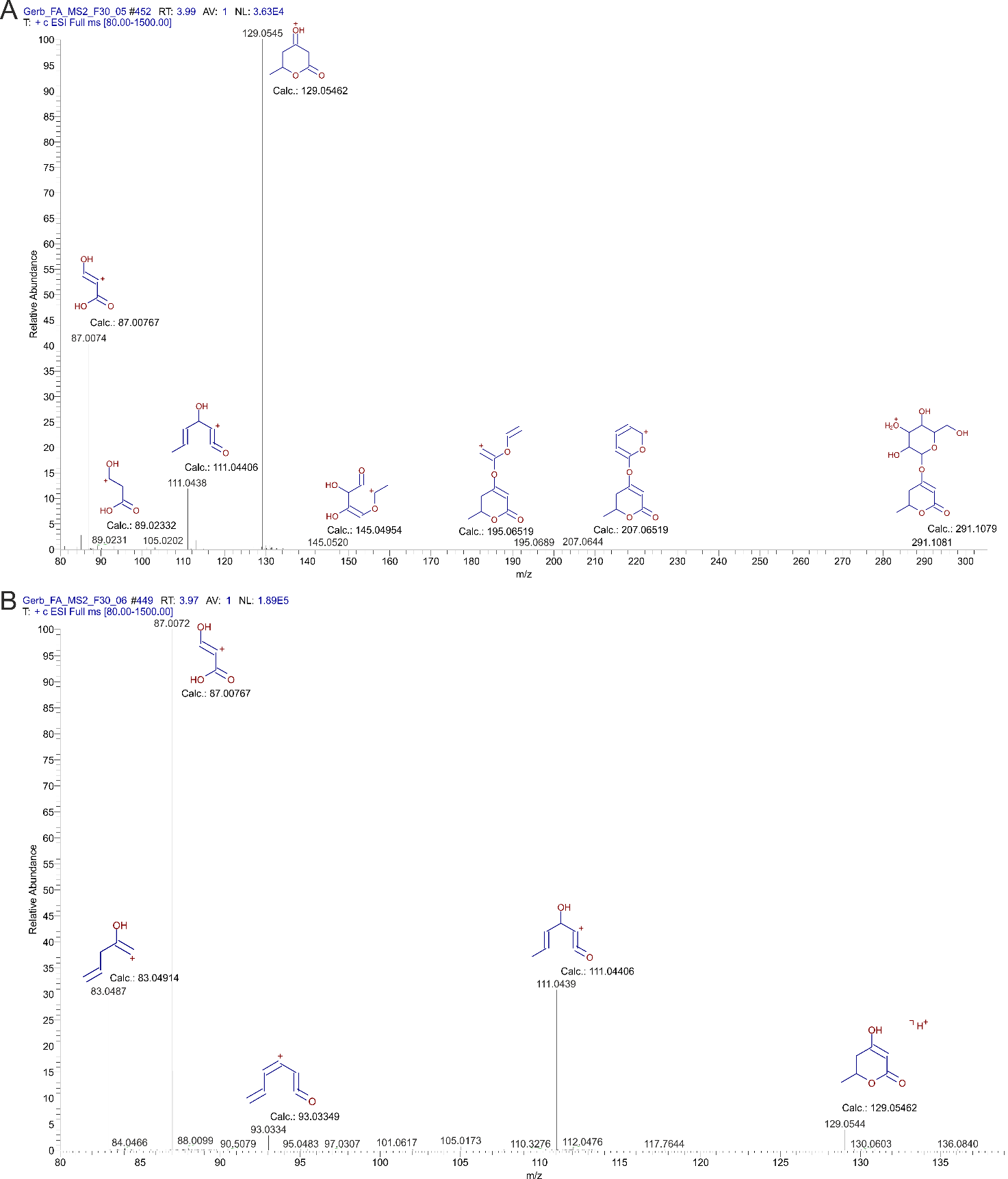
**

**Supplementary Figure S10.** MS/MS spectrum and proposed fragmentation of **(A)** gerberin at *m/z* 291.1071 [M+H]^+^ and **(B)** gerberin aglycone at *m/z* 129.0546 [M+H]^+^ (positive mode, 0.2% formic acid and methanol).


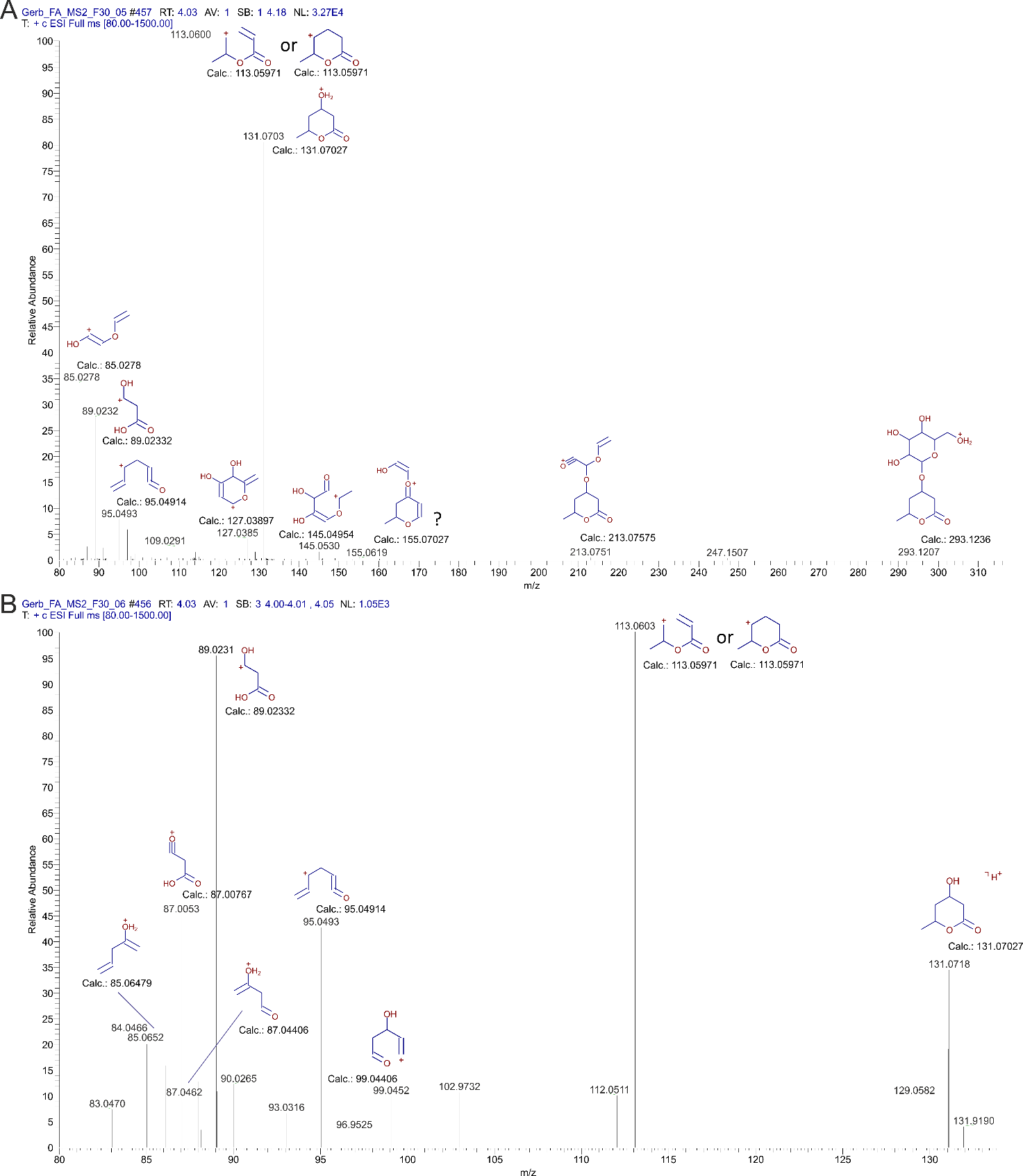


**Supplementary Figure S11.** MS/MS spectrum and proposed fragmentation of **(A)** parasorboside at *m/z* 293.1207 [M+H]^+^ and **(B)** parasorboside aglycon at *m/z* 131.0718 [M+H]^+^ (positive mode, 0.2% formic acid and methanol).

**
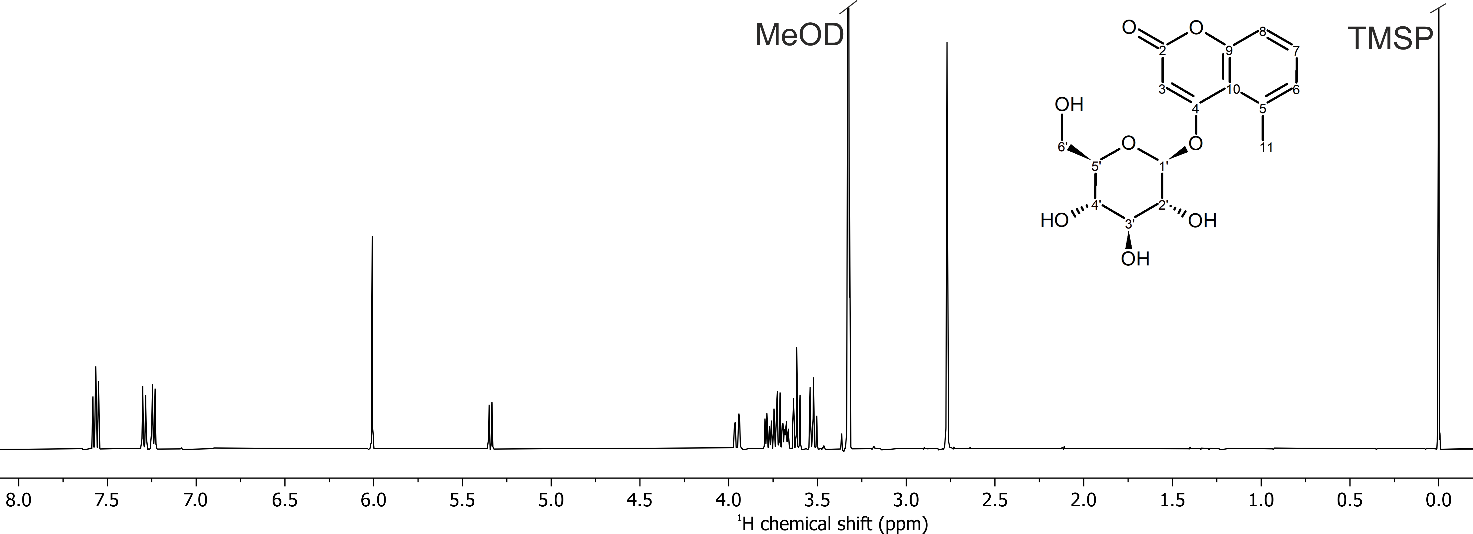
**

**Supplementary Figure S12.** ^1^H NMR spectrum of gerberinside in MeOD-D_2_O (1:1, v/v) (500MHz).


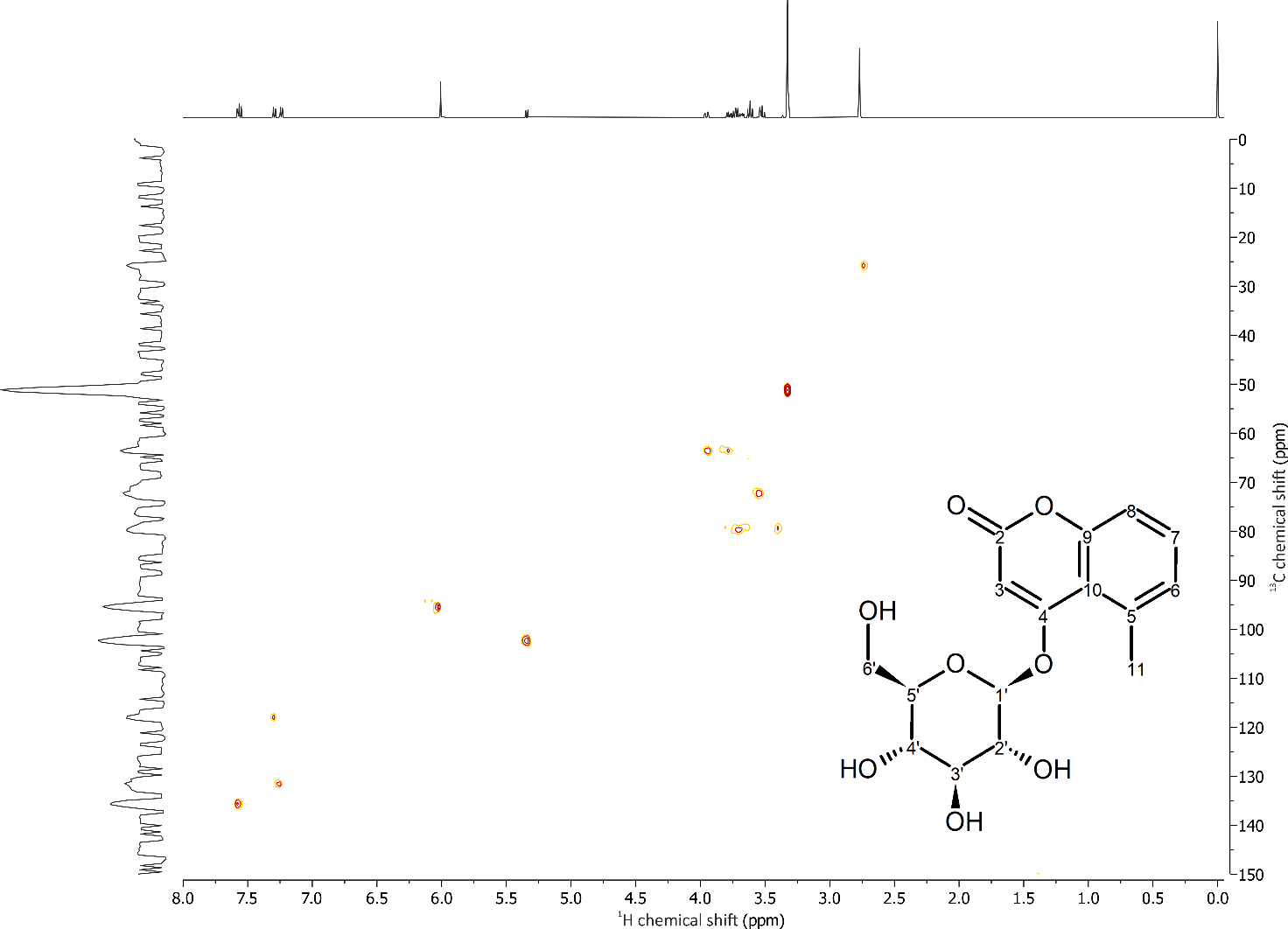


**Supplementary Figure S13.** HSQC Spectrum of gerberinside in MeOD-D_2_O (1:1, v/v).

**
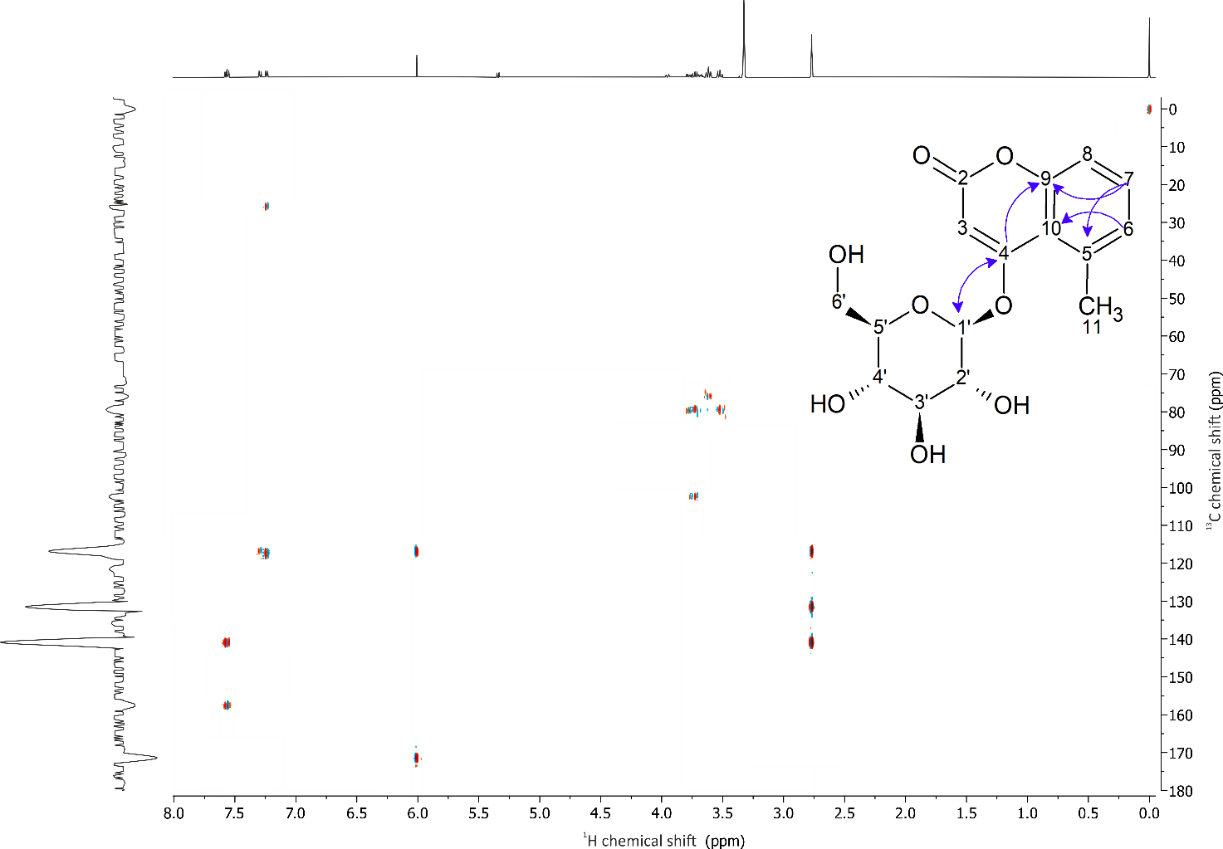
**

**Supplementary Figure S14.** HMBC Spectrum and key correlations of gerberinside in MeOD-D_2_O (1:1, v/v).


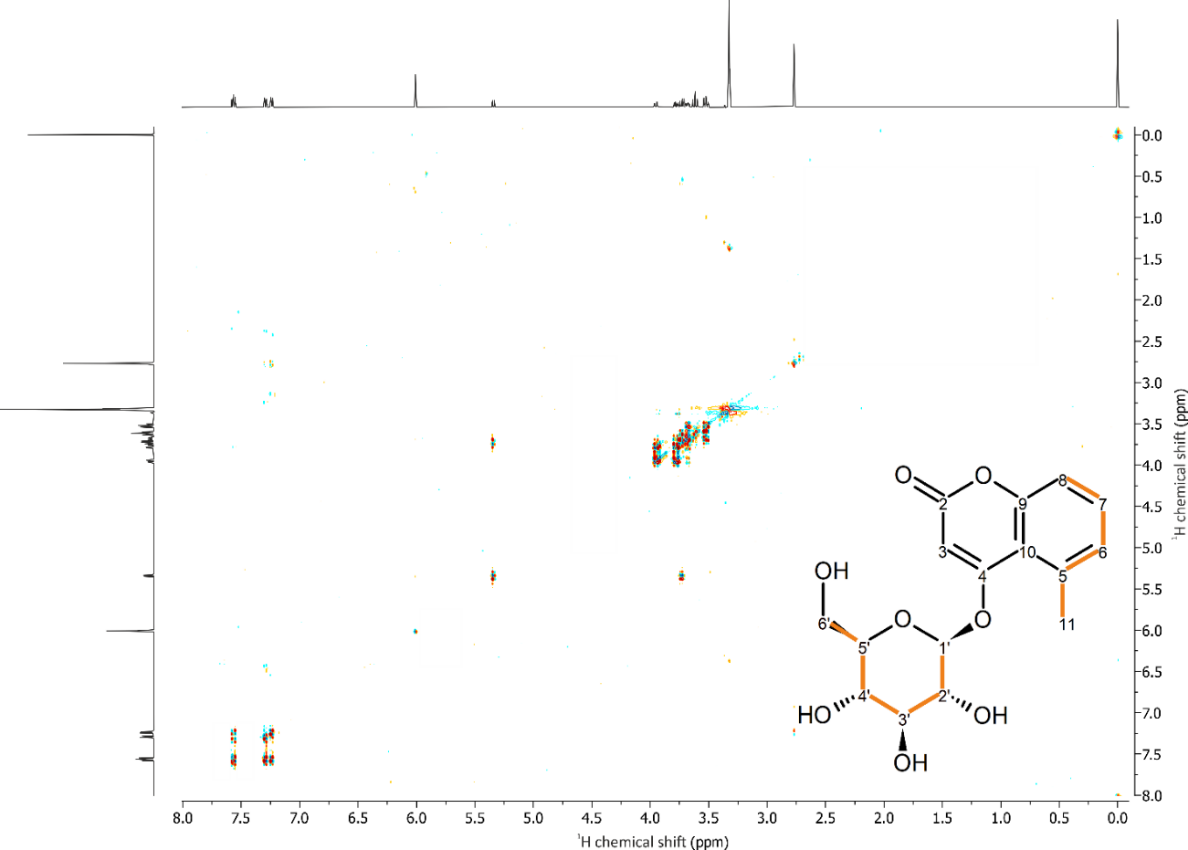


**Supplementary Figure S15.** DQF-COSY Spectrum and key correlations of gerberinside in MeOD-D_2_O (1:1, v/v).

**
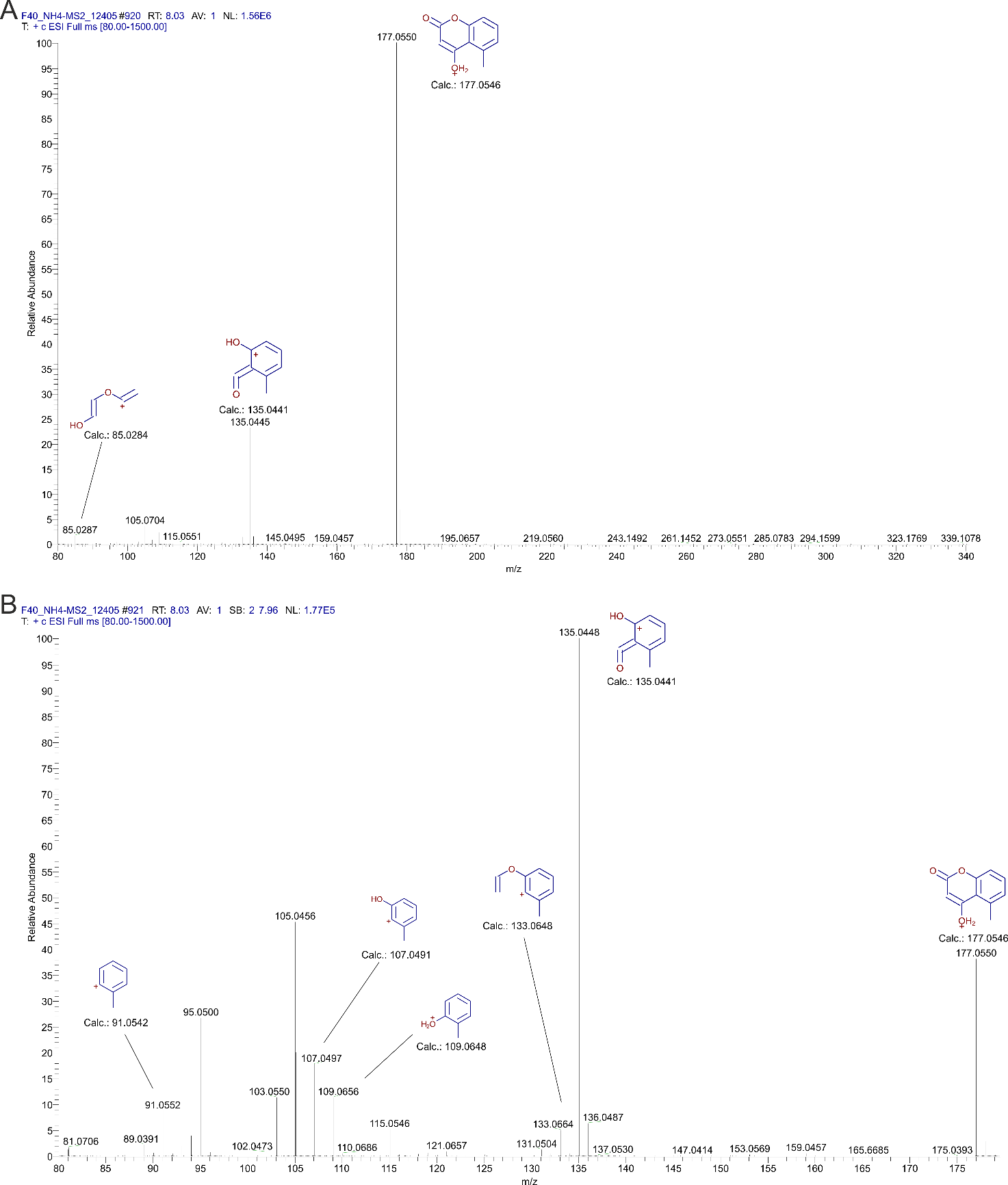
**

**Supplementary Figure S16.** MS/MS spectrum and proposed fragmentation of **(A)** gerberinside at *m/z* 339.1070 [M+H]^+^ and **(B)** gerberinside aglycone at *m/z* 177.0545 [M+H]^+^ (positive mode, 5mM ammonium formate).


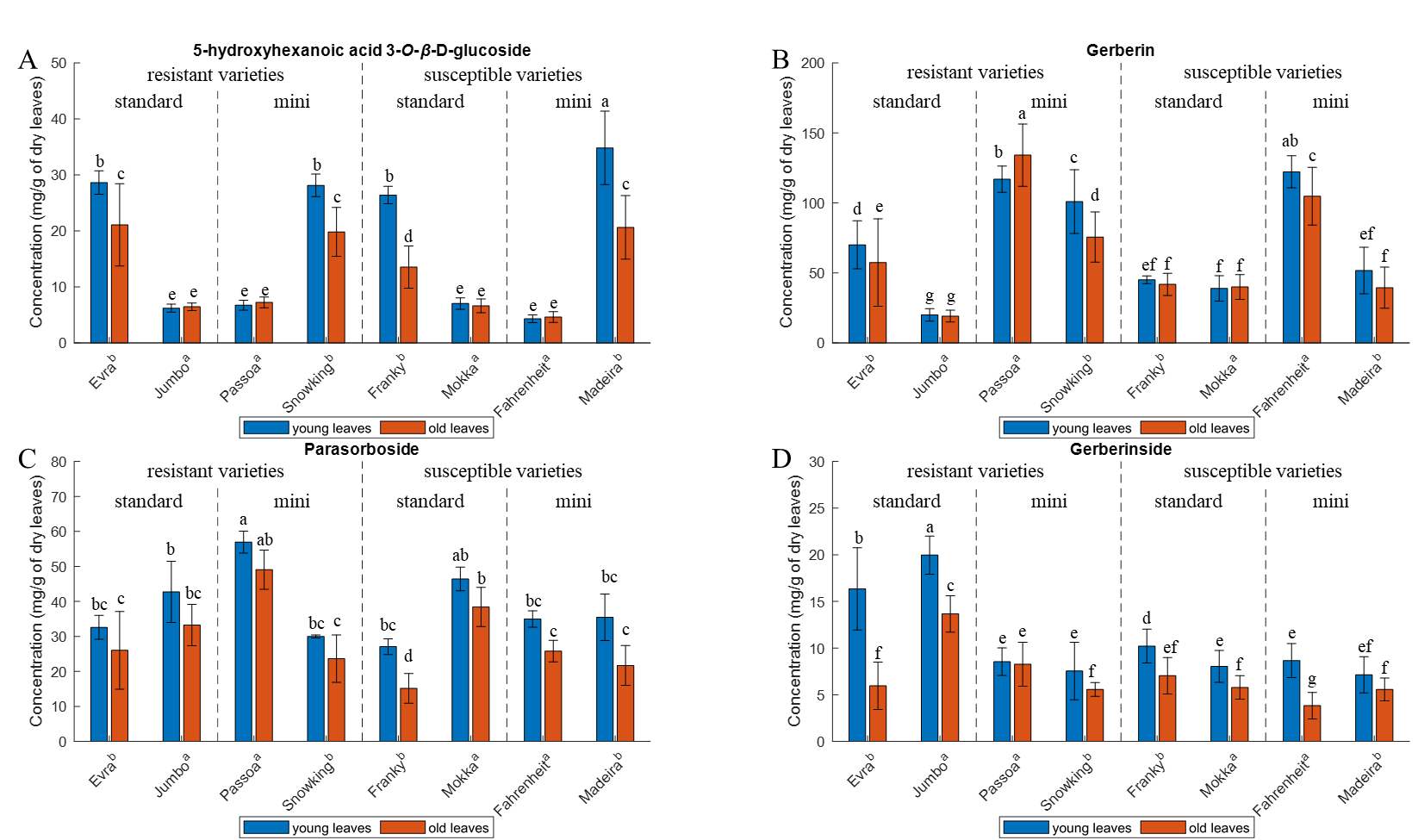


**Supplementary Figure S17.** Quantitative ^1^H NMR analysis in the leaves of varieties resistant and susceptible to powdery mildew extracted in MeOD-D_2_O (1:1, v/v). **(A)** Quantitative ^1^H NMR analysis of 5-hydroxyhexanoic acid 3-*O*-*β*-D-glucoside. **(B)** Quantitative ^1^H NMR analysis of gerberin. **(C)** Quantitative ^1^H NMR analysis of parasorboside. **(D)** Quantitative ^1^H NMR analysis of gerberinside. Error bars refer to the standard deviation (^a^N=10, ^b^N=5). Different letters on the vertical bars indicate similarities on the mean among groups. If a letter is shared, the mean is similar.
